# Supplementary material for: A comprehensive survey of cancer medicines prices, availability and affordability in Ghana
Source: PLoS One. 2023 May 3;18(5):e0279817. doi: 10.1371/journal.pone.0279817 (PMC10155977; doi:10.1371/journal.pone.0279817)
Supplement: S1 Table — (PDF) [file pone.0279817.s001.pdf]

S1 Table 2. Cancer Medicines found in Facilities, Conditions and Listing on the NEML, WHO EML, WHO EMLc

No.	International Non-Proprietary Name (INN)	Innovator Brand (OB) Name	Branded Generic Name (s)	Medicine Strength	Dosage Form	Listed in Ghana EML [14]	Listed in WHO EML [34]	Listed in WHO EMLc [35]	Chemotherapy Indication (36)
1	Abiraterone	Zytiga	-	250mg	Tab		x		Hormones for prostate cancer.
2	Anastrozole	Arimidex	-	1mg	Tab		x		Hormones and antagonist for breast cancer.
3	Bevacizumab	Avastin,	-	400mg	Vial				Colorectal
4	Bicalutamide	Casodex	-	50mg	Tab	x	x		Hormones and antagonist for prostate cancer.
5	Bicalutamide	Casodex	-	150mg	Tab				Hormones and antagonist for prostate cancer.
6	Bleomycin	Bleo-Kyowa	Bleowel, Bleocel	15 IU PFR	Vial		x	x	Natural product for testis, cervical ovarian, Hodgkin's disease, non-Hodgkin's lymphoma.
7	Bortezomib	Velcade	Neomib	3.5mg	Vial		x		Miscellaneous agent for Multiple myeloma and mantle cell lymphoma.
8	Capecitabine	Xeloda	-	500mg	Tab		x		Anti-metabolite for breast, colon, esophageal, stomach, pancreas, premalignant skin lesion (topical), head and neck.

9	Carboplatin	Paraplatin	Carbotin, Carbotinol, Kemocarb	150mg	Vial	x	x	Alkylating agent for testicular, ovarian, bladder, esophageal, lung, colon, breast, brain, neuroblastoma, head, and neck.
10	Carboplatin	Paraplatin	Carbotin, Carbotinol, Kemocarb	450mg	Vial	x	x	Alkylating agent for testicular, ovarian, bladder, esophageal, lung, colon, breast, brain, neuroblastoma, head, and neck.
11	Chlorambucil	Leukeran	Celkeran, Chloramax	2mg	Tab	x		Alkylating agent for chronic lymphocytic leukemia, macroglobulinemia, Hodgkin lymphoma, and non-Hodgkin lymphoma
12	Cisplatin	Platinol	Cistero-10, Abiplatin, Kemoplat	10mg/10 ml	Vial	x	x	Alkylating agent for testicular, ovarian, bladder, esophageal, lung, head and neck, colon, breast, cervical, mesothelioma, brain tumors and neuroblastoma.

13	Cisplatin	Platinol	Cistero-50, Kemoplat, Celplat	50mg	Vial	x	x	x	Alkylating agent for testicular, ovarian, bladder, esophageal, lung, head and neck, colon, breast, cervical, mesothelioma, brain tumors and neuroblastoma.
14	Cyclophosphamide	Endoxan, Cytoxan	Cycloxan, Phoxelon	50mg	Tabs	x	x	x	Alkylating Agent for acute and chronic lymphocytic leukemia, Hodgkin's disease, multiple myeloma, ovarian, breast, small cell lung, neuroblastoma, and sarcoma.
15	Cyclophosphamide	Endoxan, Cytoxan	Phoxelon-500, Cyphos	500mg	Vial	x	x	x	Alkylating Agent for acute and chronic lymphocytic leukemia, Hodgkin's disease, multiple myeloma, ovarian, breast, small cell lung, neuroblastoma, and sarcoma.
16	Cyclophosphamide	Endoxan, Cytoxan	Cyphos	1g	Vial		x	x	Alkylating Agent for acute and chronic lymphocytic leukemia, Hodgkin's disease,

								multiple myeloma, ovarian, breast, small cell lung, neuroblastoma, and sarcoma.
17	Cytarabine	Cytosar-U	Cytalon-100	100mg	Vial	x	x	Anti-metabolite for acute myelogenous and acute lymphocytic leukemia and non-Hodgkin’s lymphoma.
18	Dacarbazine	DTIC-Dome	Celdaz, Dacarex	200mg	Vial			Alkylating agent for malignant melanoma, Hodgkin’s disease, and soft-tissue melanoma.
19	Dacarbazine	DTIC-Dome	Celdaz-500	500mg	Vial			Alkylating agent for malignant melanoma, Hodgkin’s disease, and soft-tissue melanoma.
20	Dactinomycin/ Actinomycin D	Cosmegen	Dacilon	0.5mg	Vial	x	x	Natural product for choriocarcinoma, Wilms’ tumor, rhabdomyosarcoma, testis, Kaposi’s sarcoma, Ewing's sarcoma, trophoblastic neoplasm, and ovarian.
21	Daunorubicin	**C**erubidine	Daunotec	20mg	Vial			Natural product for acute and chronic myelogenous leukemia, and acute

									lymphocytic leukemia, and Kaposi's sarcoma.
22	Docetaxel Trihydrate	Taxotere	Docetero-20	20mg	Vial		x		Natural products for ovarian, breast, lung, prostate, bladder, stomach, head, and neck cancer
23	Docetaxel Trihydrate	Taxotere	Daxotel, Docetero-80, Docetaxel Sandoz	80mg	Vial				Natural products for ovarian, breast, lung, prostate, bladder, stomach, head, and neck cancer
24	Docetaxel Trihydrate	Taxotere	-	120mg	Vial				Natural products for ovarian, breast, lung, prostate, bladder, stomach, head, and neck cancer
25	Doxorubicin HCL	Caelyx, Adriblastina RD, Adriblastina CSV	-	10mg	Vial	x	x	x	Natural product for soft-tissue, osteogenic, and other sarcoma, Breast, bladder, Kaposi's sarcoma, Hodgkin’s disease, non-Hodgkin’s lymphoma, acute lymphocytic leukemia, breast, genitourinary, thyroid, lung, stomach,

									neuroblastoma and other childhood and adult sarcomas.
26	Doxorubicin HCL	Caelyx, Adriblastina RD, Adriblastina CSV	Doxinyl -50, Doxorubicine HCl Sandoz	50mg	Vial		x	x	Natural product for soft-tissue, osteogenic, and other sarcoma, Breast, bladder, Kaposi's sarcoma, Hodgkin’s disease, non-Hodgkin’s lymphoma, acute lymphocytic leukemia, breast, genitourinary, thyroid, lung, stomach, neuroblastoma and other childhood and adult sarcomas.
27	Epirubicin	Pharmorubicin	Epiget-50, Epiruba	50mg	Vial				Breast
28	Etoposide	Vepesid, Etopophos	Posid, Etopa, Etovel, Oncosid-100	100mg/5 ml	Vial	x	x	x	Natural product for testis, lung, breast cancer, Hodgkin’s disease, non-Hodgkin’s lymphomas; acute myelogenous leukemia, Kaposi’s sarcoma, neuroblastoma, and ovarian.
29	Exemestane	Aromasin	-	25mg	Tab				Hormones and antagonist for breast cancer.

30	Filgrastim	Neupogen, Zarzio, Nivestim, Accofil	-	300mcg	Vial		x	x	Hormonal Immune modulator for prophylaxis in patients at high risk for developing or have developed febrile neutropenia associated with myelotoxic chemotherapy.
31	Fluorouracil	Fluroblastin,	Raciwel	50mg/ml	Vial		x	x	Anti-metabolite for breast, colon, esophageal, stomach, pancreas, cervical, premalignant skin lesion (topical), head and neck.
32	Fluorouracil	Fluroblastin,	Raciwel, Fluracil, 5-flucel	500mg	Vial				Anti-metabolite for breast, colon, esophageal, stomach, pancreas, cervical, premalignant skin lesion (topical), head and neck.
33	Gemcitabine	Gemzar,	Gemget-1000, Gemwel	1000mg	Vial		x		Antimetabolite for pancreatic, ovarian, lung, testicular, breast, and bladder.
34	Goserelin	Zoladex	-	3.6mg	Vial	x			Adjuvant for hormone therapy

35	Goserelin	Zoladex	-	10.8mg	Vial	x			Adjuvant for hormone therapy
36	Hydroxy Urea (hydroxy carbamide)	Hydrea, Siklos	-	250mg	Tabs		x	x	Miscellaneous agent for chronic myelogenous leukemia, polycythemia, cervical, and essential thrombocytosis.
37	Ifosfamide+ Mesna	Haloxan 2G with Uromitexan	-	1g	Vial		x	x	Alkylating Agent for non-Hodgkin’s lymphoma, multiple myeloma, neuroblastoma, breast, ovary, lung cancer, Wilms’ tumor, cervix, testis, soft-tissue sarcoma, bladder, muscles, and bones.
38	Imatinib	Gleevec, Glivec	Veenat-100	100mg	Tab		x	x	Miscellaneous agent for chronic myelogenous leukemia, gastrointestinal stromal tumors, hyper eosinophilia syndrome.
39	Imatinib	Gleevec, Glivec	-	400mg	Tab		x	x	Miscellaneous agent for chronic myelogenous leukemia, gastrointestinal stromal tumors, hyper eosinophilia syndrome.
40	Irinotecan	Campto	Irinotel	100mg/5 ml	Vial		x	x	Natural product for colon, and small cell lung.

41	L-Asparaginase	Spectrila	Bionase	10,000iu	Vial		x	x	Natural product for acute lymphoblastic leukemia.
42	Lenalidomide	Revlimid	Lenalid-10	10mg	Cap				Miscellaneous agent (immune modulator) for Myelodysplasia (5q− syndrome), multiple myeloma.
43	Leuprolide Acetate	Prostap, Lupron	-	3.75mg	Vial	x			Hormone and antagonist for prostate and breast.
44	Leuprolide Acetate	Prostap, Lupron	Luprova	11.25mg	Vial				Hormone and antagonist for prostate and breast.
45	Liposomal Doxorubicin	Myocet, Doxil, Caelyx	-	20mg	Vial				Natural product for soft-tissue, osteogenic, and other sarcoma, Hodgkin's disease, non-Hodgkin's lymphoma, acute leukemia, breast, genitourinary, thyroid, lung, and stomach cancer, neuroblastoma, Kaposi's sarcoma, and other childhood and adult sarcomas.
46	Liposomal Doxorubicin	Myocet, Doxil, Caelyx	-	50mg	Vial		x	x	Natural product for soft-tissue, osteogenic, and other sarcoma, Hodgkin's disease, non-Hodgkin's lymphoma, acute

									leukemia, breast, genitourinary, thyroid, lung, and stomach cancer, neuroblastoma, Kaposi's sarcoma, and other childhood and adult sarcomas.
47	Melphalan	Alkeran, Evomela	Alkacel-2	2mg	Tab		x		Alkylating agent for multiple myeloma, ovarian, melanoma, and AL amyloidosis.
48	Mercaptopurine	Puri-Nethol, Xaluprine		50mg	Tab		x	x	Anti-metabolite for acute lymphocytic and chronic myelogenous leukemia, small cell non-Hodgkin’s lymphoma, Crohn's disease, and ulcerative colitis.
49	Mercaptopurine	Puri-Nethol, Xaluprine	-	150mg	Tab				Anti-metabolite for acute lymphocytic and chronic myelogenous leukemia, small cell non-Hodgkin’s lymphoma, Crohn's disease, and ulcerative colitis.
50	Methotrexate	Methofill, Metoject PEN,	Biotrexate	2.5mg	Tab	x	x	x	Antimetabolite for acute lymphocytic leukemia; choriocarcinoma; breast,

		Nordimet, Zlatal						head, neck and lung cancers; osteogenic sarcoma; bladder cancer.
51	Methotrexate	Methofill, Metoject PEN, Nordimet, Zlatal	Methocel-50	50mg	Vial	x	x	Antimetabolite for acute lymphocytic leukemia; choriocarcinoma; breast, head, neck and lung cancers; osteogenic sarcoma; bladder cancer.
52	Mitomycin	Mitocin		10mg	Vial			Natural product for stomach, anal, breast, superficial bladder tumors and lung cancer.
53	Oxaliplatin	Eloxatin		100mg	Vial	x	x	Alkylating agent for testicular, ovarian, bladder, esophageal, lung, colorectal, breast cancer head and neck.
54	Paclitaxel	Taxol, Abraxane	Intaxel, Ataxil, Paclitec-100, Pacliwel, Paclitec-100, Paclitaxel Sandoz	100mg	Vial			Natural products for ovarian, breast, lung, prostate, bladder, esophageal, Kaposi sarcoma, cervical, and pancreatic, head and neck cancer.
55	Sorafenib	Nexavar	Soranib, Orib, Sorafenat	200mg	Tab			Miscellaneous agent for renal, primary kidney cancer, advanced primary

								liver cancer, FLT3-ITD positive AML and radioactive iodine resistant advanced thyroid carcinoma.
56	Tamoxifen	Kessar 10, Nolvadex, Soltamox, Tamoxen	Tamoxifen-Teva	10mg	Tab	x		Hormones and antagonist for breast cancer.
57	Tamoxifen	Nolvadex- D / Kessar 20, Nolvadex, Soltamox, Tamoxen	Cytotam	20mg	Tab	x		Hormones and antagonist for breast cancer.
58	Thalidomide	Talidex	Thalix-50	50mg	Cap	x		Miscellaneous agent (immune modulator) for multiple myeloma.
59	Thalidomide	Talidex	Thalix-100	100mg	Cap			Miscellaneous agent (immune modulator) for multiple myeloma.
60	Trastuzumab	Herceptin,	-	600mg	Vial	x		Breast and stomach
61	Triptorelin Acetate	Decapeptyl	-	3.75mg	Vial			Prostate
62	Vinblastine	Velban	Chemoblast	10mg	Vial	x	x	Natural products for Hodgkin’s disease, non-Hodgkin’s lymphoma, non-small cell lung,

									bladder, brain, melanoma, and testis.
63	Vincristine	Oncovin, Vincasar, Marqibo	Biocristine-AQ, Vincristine Medcrist, Vinlon-1, Vincristine Micristin, Cytocristin	1mg	Vial	x	x	x	Natural products for acute lymphocytic leukemia, acute myeloid leukemia, neuroblastoma, Wilms' tumor, rhabdomyosarcoma; Hodgkin's disease; non-Hodgkin's lymphoma, and small cell lung.
64	Vinorelbine	Navelbine	Vinelbine	50mg	Vial		x	x	Natural products for breast and non-small cell lung.
65	Zoledronic Acid	Zometa	Zoldron, Zelodro-Denk	4mg/5ml	vial		x		Adjuvant for bone diseases.
