# Supplementary material for: A comprehensive survey of cancer medicines prices, availability and affordability in Ghana
Source: PLoS One. 2023 May 3;18(5):e0279817. doi: 10.1371/journal.pone.0279817 (PMC10155977; doi:10.1371/journal.pone.0279817)
Supplement: S2 Table — (PDF) [file pone.0279817.s002.pdf]

S2 Table 5a. Price Variations of Cancer Medicine(s) in Public Hospitals:

No.	Medicine Name	Medicine Strength	Dosage Form	Target Pack Size	Medicine Type	Minimum (USD)	Maximum (USD)	Cost Differential between Min and Max (%)	Price Ratio
1	Bicalutamide (Casodex)	150mg	tabs	1	OB	4.81	99.12	95.15	20.60
2	Carboplatin (Carbotin, Carbotinol, Kemocarb)	150mg	vial	1	LPG	21.48	32.05	32.99	1.49
3	Carboplatin (Carbotin, Carbotinol, Kemocarb)	450mg	vial	1	LPG	58.15	79.63	26.97	1.37
4	Cisplatin (Cistero-50, Kemoplat, Celplat)	50mg	vial	1	LPG	8.26	15.53	46.81	1.88
5	Cyclophosphamide (Phoxelon-500, Cyphos)	500mg	vial	1	LPG	2.64	3.30	20.00	1.25
6	Doxorubicin HCL (Doxinyl -50, Doxorubicine HCl Sandoz)	50mg	vial	1	LPG	14.04	16.52	15.00	1.18
7	Etoposide (Posid, Etopa, Etovel, Oncosid-100)	100mg	vial	1	LPG	5.62	6.94	19.05	1.24
8	Fluorouracil (Raciwel, Fluracil, 5-flucel)	500mg	vial	1	LPG	1.98	2.31	14.29	1.17
9	Goserelin (Zoladex)	10.8mg	vial	1	OB	223.02	289.35	22.92	1.30
10	Methotrexate (Methocel-50)	50mg	vial	1	LPG	5.45	5.95	8.33	1.09
11	Paclitaxel (Intaxel, Ataxil, Paclitec-100, Pacliwel,	100mg	vial	1	LPG	32.21	37.17	13.33	1.15

	Paclitec-100, Paclitaxel Sandoz)								
12	Vincristine (Biocristine-AQ, Vincristine Medcrist, Vinlon-1, Vincristine Micristin, Cytocristin)	1mg	vial	1	LPG	2.81	2.97	5.56	1.06
