# Supplementary material for: A comprehensive survey of cancer medicines prices, availability and affordability in Ghana
Source: PLoS One. 2023 May 3;18(5):e0279817. doi: 10.1371/journal.pone.0279817 (PMC10155977; doi:10.1371/journal.pone.0279817)
Supplement: S4 Table — (PDF) [file pone.0279817.s004.pdf]

S4 Table 5c. This is the S4 Table 5c. Price Variations of Cancer Medicine(s) in Private Pharmacies

No.	Medicine Name	Medicine Strength	Dosage Form	Target Pack Size	Medicine Type	Minimum (USD)	Maximum (USD)	Cost Differential between Min and Max (%)	Price Ratio
1	Abiraterone (Zytiga)	250mg	tabs	1	OB	4.79	6.20	22.67	1.29
2	Anastrozole (Arimidex)	1mg	tabs	1	OB	1.07	1.72	37.46	1.60
3	Bleomycin (Bleowel, Bleocel)	15 IU PFR	vial	1	LPG	23.95	28.91	17.14	1.21
4	Capecitabine (Xeloda)	500mg	tabs	1	OB	2.15	2.81	23.53	1.31
5	Carboplatin (Carbotin, Carbotinol, Kemocarb)	150mg	vial	1	LPG	16.52	82.68	80.02	5.01
6	Carboplatin (Carbotin, Carbotinol, Kemocarb)	450mg	vial	1	LPG	24.12	79.63	69.71	3.30
7	Chlorambucil (Celkeran, Chloramax)	2mg	tabs	1	LPG	1.98	7.10	72.09	3.58
8	Cisplatin (Cistero-10, Abiplatin, Kemoplat)	10mg	vial	1	LPG	14.87	19.33	23.08	1.30
9	Cisplatin (Cistero-50, Kemoplat, Celplat)	50mg	vial	1	LPG	13.22	18.17	27.27	1.38
10	Cyclophosphamide (Cyphos)	1g	vial	1	LPG	3.63	18.50	80.36	5.09
11	Cyclophosphamide (Cycloxan, Phoxelon)	50mg	tabs	1	LPG	0.26	3.47	92.38	13.13
12	Cyclophosphamide (Phoxelon-500, Cyphos)	500mg	vial	1	LPG	2.97	4.96	40.00	1.67

13	Cytarabin (Cytalon-100)	100mg	vial	1	LPG	9.91	17.35	42.86	1.75
14	Docetaxel Trihydrate (Docetero-20)	20mg	vial	1	LPG	42.29	46.26	8.57	1.09
15	Docetaxel Trihydrate (Daxotel, Docetero-80, Docetaxel Sandoz)	80mg	vial	1	LPG	92.51	111.51	17.04	1.21
16	Doxorubicin HCL (Doxinyl - 50, Doxorubicine HCl Sandoz)	50mg	vial	1	LPG	13.22	18.17	27.27	1.38
17	Etoposide (Posid, Etopa, Etovel, Oncosid-100)	100mg	vial	1	LPG	6.44	9.09	29.09	1.41
18	Exemestane (Aromasin)	25mg	tabs	1	OB	1.38	9.17	84.97	6.65
19	Fluorouracil (Raciwel, Fluracil, 5-flucel)	500mg	vial	1	LPG	1.90	3.30	42.50	1.74
20	Gemcitabine (Gemget-1000, Gemwel)	1000mg	vial	1	LPG	94.99	132.16	28.13	1.39
21	Goserelin (Zoladex)	3.6mg	vial	1	OB	109.28	194.11	43.70	1.78
22	Goserelin (Zoladex)	10.8mg	vial	1	OB	264.32	341.65	22.63	1.29
23	Hydroxyurea (Hydrea, Siklos)	250mg	tabs	1	OB	0.41	0.83	50.00	2.00
24	Leuprolide Acetate (Luprova)	11.25mg	vial	1	LPG	173.96	181.72	4.27	1.04
25	Melphalan (Alkacel-2)	2mg	tabs	1	LPG	2.51	3.30	24.00	1.32
26	Methotrexate (Biotrexate)	2.5mg	tabs	1	LPG	0.12	0.38	69.57	3.29
27	Methotrexate (Biotrexate Methocel-50)	50mg	vial	1	LPG	6.61	8.26	20.00	1.25
28	Mitomycin	10mg	vial	1	LPG	32.21	43.12	25.29	1.34

29	Paclitaxel (Intaxel, Ataxil, Paclitec-100, Pacliwel, Paclitec-100, Paclitaxel Sandoz)	100mg	vial	1	LPG	23.95	39.65	39.58	1.66
30	Sorafenib (Soranib, Orib, Sorafenat)	200mg	tabs	1	LPG	1.82	4.30	57.69	2.36
31	Tamoxifen (Cytotam)	20mg	tabs	1	LPG	0.33	0.79	58.33	2.40
32	Thalidomide (Thalix-50)	50mg	caps	1	LPG	1.16	55.51	97.92	48.00
33	Thalidomide (Thalix-100)	100mg	cap	1	LPG	1.82	2.48	26.67	1.36
34	Trastuzumab	600mg	vial	1	OB	563.75	598.68	5.84	1.06
35	Vincristine (Biocristine-AQ, Vincristine Medcrist, Vinlon-1, Vincristine Micristin, Cytocristin)	1mg	vial	1	LPG	2.97	6.61	55.00	2.22
36	Zoledronic Acid (Zoldron, Zelodro-Denk)	4mg/5ml	vial	1	LPG	39.32	54.52	27.88	1.39
