# Supplementary material for: A comprehensive survey of cancer medicines prices, availability and affordability in Ghana
Source: PLoS One. 2023 May 3;18(5):e0279817. doi: 10.1371/journal.pone.0279817 (PMC10155977; doi:10.1371/journal.pone.0279817)
Supplement: S5 Table — (PDF) [file pone.0279817.s005.pdf]

S5 Table 6a. MPR of Cancer Medicines in Public Hospitals

	Medicine Name	Medicine Strength	Dosage Form	Medicine Type	2020 Median Price (USD)	2015 MSH Price (USD)	Deflated local prices from 2020 (USD)	Median Price Ratio (MPR)
1	Bicalutamide (Casodex)	50mg	tabs	OB	1.43	0.23	0.22	0.93
2	Bicalutamide	50mg	tabs	LPG	0.48	0.23	0.07	0.31
3	Bicalutamide (Casodex)	150mg	tabs	OB	51.97	1.03	7.94	7.71
4	Bicalutamide	150mg	tabs	LPG	0.53	1.03	0.08	0.08
5	Bleomycin (Bleowel, Bleocel)	15 IU PFR	vial	LPG	18.17	12.32	2.77	0.23
6	Carboplatin	150mg	vial	LPG	26.76	16.01	4.09	0.26
7	Carboplatin (Carbotin, Carbotinol, Kemocarb)	450mg	vial	LPG	68.89	40.32	10.52	0.26
8	Carboplatin (Carbotin, Carbotinol, Kemocarb)	2mg	tabs	LPG	1.73	0.75	0.26	0.36
9	Cisplatin (Cistero-10, Abiplatin, Kemoplat)	10mg	vial	LPG	11.73	5.03	1.79	0.36
10	Cisplatin (Cistero-50, Kemoplat, Celplat)	50mg	vial	LPG	11.89	7.25	1.82	0.25
11	Cyclophosphamide (Cyphos)	1g	vial	LPG	5.95	8.27	0.91	0.11
12	Cyclophosphamide (Phoxelon-500, Cyphos)	500mg	vial	LPG	2.97	5.24	0.45	0.09
13	Dacarbazine (Celdaz, Dacarex)	200mg	vial	LPG	14.04	6.81	2.14	0.31

14	Docetaxel Trihydrate (Docetero-20)	20mg	vial	LPG	33.04	40.50	5.05	0.12
15	Docetaxel Trihydrate (Daxotel, Docetero-80, Docetaxel Sandoz)	80mg	vial	LPG	84.25	47.97	12.87	0.27
16	Doxorubicin HCL (Doxinyl -50, Doxorubicine HCl Sandoz)	50mg	vial	LPG	16.19	5.41	2.47	0.46
17	Epirubicin (Epiget-50, Epiruba)	50mg	vial	LPG	46.26	21.68	7.06	0.33
18	Etoposide (Posid, Etopa, Etovel, Oncosid-100)	100mg	vial	LPG	6.28	2.02	0.96	0.48
19	Filgrastim (Neupogen, Zarzio, Nivestim, Accofil)	300mcg	vial	OB	3.63	75.57	0.55	0.01
20	Fluorouracil (Raciwel)	50mg	vial	LPG	1.16	1.22	0.18	0.14
21	Fluorouracil (Raciwel, Fluracil, 5-flucel)	500mg	vial	LPG	2.15	0.26	0.33	1.25
22	Gemcitabine (Gemget-1000, Gemwel)	1000mg	vial	LPG	90.86	25.27	13.87	0.55
23	Ifosfamide + Mesna (Haloxan 2G with Uromitexan)	1g	vial	OB	9.09	26.71	1.39	0.05
24	Oxaliplatin	100mg	vial	LPG	66.08	74.77	10.09	0.13
25	Paclitaxel (Intaxel, Ataxil, Paclitec-100, Pacliwel, Paclitec-100, Paclitaxel Sandoz)	100mg	vial	LPG	33.54	11.08	5.12	0.46

26	Tamoxifen (Tamoxifen-Teva)	20mg	tabs	LPG	8.26	0.12	1.26	10.15
27	Vincristine (Biocristine-AQ, Vincristine Medcrist, Vinlon-1, Vincristine Micristin, Cytocristin)	1mg	vial	LPG	2.89	2.54	0.44	0.17
28	Vinorelbine	50mg	vial	LPG	74.34	29.01	11.35	0.39
29	Zoledronic Acid	4mg/5ml	vial	LPG	38.00	23.45	5.80	0.25
