# Supplementary material for: A comprehensive survey of cancer medicines prices, availability and affordability in Ghana
Source: PLoS One. 2023 May 3;18(5):e0279817. doi: 10.1371/journal.pone.0279817 (PMC10155977; doi:10.1371/journal.pone.0279817)
Supplement: S6 Table — (PDF) [file pone.0279817.s006.pdf]

S6 Table 6b. MPR of Cancer Medicines in Private Hospitals

	Medicine Name Generic	Medicine Strength	Dosage Form	Medicine Type	2020 Median Price (USD)	2015 MSH Price (USD)	Deflated local prices from 2020 (USD)	Median Price Ratio (MPR)
1	Anastrozole (Arimidex)	1mg	tabs	OB	0.71	0.53	0.11	0.21
2	Bleomycin (Bleowel, Bleocel)	15 IU PFR	vial	LPG	46.26	12.32	7.06	0.57
3	Capecitabine (Xeloda)	500mg	tabs	OB	2.62	1.67	0.40	0.24
4	Carboplatin (Carbotin, Carbotinol, Kemocarb)	450mg	vial	LPG	99.12	40.32	15.14	0.38
5	Cisplatin (Cistero-50, Kemoplat, Celplat)	50mg	vial	LPG	15.69	7.25	2.40	0.33
6	Cyclophosphamide (Cyphos)	1g	vial	LPG	7.85	8.27	1.20	0.14
7	Cyclophosphamide (Endoxan, Cytoxan)	50mg	tabs	OB	0.41	0.30	0.06	0.21
8	Docetaxel Trihydrate (Daxotel, Docetero-80, Docetaxel Sandoz)	80mg	vial	LPG	132.57	47.97	20.24	0.42
9	Doxorubicin HCL (Doxinyl -50, Doxorubicine HCl Sandoz)	50mg	vial	LPG	14.12	5.41	2.16	0.40
10	Epirubicin (Pharmorubicin)	50mg	vial	OB	85.90	21.68	13.12	0.61
11	Etoposide (Posid, Etopa, Etovel, Oncosid-100)	100mg	vial	LPG	16.52	2.02	2.52	1.25
12	Fluorouracil (Raciwel)	500mg	vial	LPG	4.13	0.26	0.63	2.41
13	Gemcitabine (Gemget-1000, Gemwel)	1000mg	vial	LPG	165.20	25.27	25.23	1.00
14	Oxaliplatin	100mg	vial	LPG	132.16	74.77	20.18	0.27

15	Paclitaxel (Intaxel, Ataxil, Paclitec-100, Pacliwel, Paclitec-100, Paclitaxel Sandoz)	100mg	vial	LPG	49.56	11.08	7.57	0.68
16	Vincristine (Biocristine-AQ, Vincristine Medcrist, Vinlon-1, Vincristine Micristin, Cytocristin)	1mg	vial	LPG	2.97	2.54	0.45	0.18
17	Zoledronic Acid (Zoldron, Zelodro-Denk)	4mg/5ml	vial	LPG	82.60	23.45	12.61	0.54
