# Supplementary material for: A comprehensive survey of cancer medicines prices, availability and affordability in Ghana
Source: PLoS One. 2023 May 3;18(5):e0279817. doi: 10.1371/journal.pone.0279817 (PMC10155977; doi:10.1371/journal.pone.0279817)
Supplement: S7 Table — (PDF) [file pone.0279817.s007.pdf]

S7 Table 6c. MPR of Cancer Medicines in Private Pharmacies

No.	Medicine Name	Medicine Strength	Dosage Form	Medicine Type	2020 Median Price (USD)	2015 MSH Price (USD)	Deflated local prices from 2020 (USD)	Median Price Ratio (MPR)
1	Anastrozole (Arimidex)	1mg	tabs	OB	1.14	0.53	0.17	0.33
2	Anastrozole	1mg	tabs	LPG	0.50	0.53	0.08	0.14
3	Bicalutamide (Casodex)	50mg	tabs	OB	1.58	0.23	0.24	1.03
4	Bicalutamide (Casodex)	150mg	tabs	OB	3.47	1.03	0.53	0.51
5	Bleomycin (Bleowel, Bleocel)	15 IU PFR	vial	LPG	26.43	12.32	4.04	0.33
6	Capecitabine (Xeloda)	500mg	tabs	OB	2.64	1.67	0.40	0.24
7	Carboplatin (Carbotin, Carbotinol, Kemocarb)	150mg	vial	LPG	27.84	16.01	4.25	0.27
8	Carboplatin (Carbotin, Carbotinol, Kemocarb)	450mg	vial	LPG	74.34	40.32	11.35	0.28
9	Chlorambucil (Leukeran)	2mg	tabs	OB	2.51	0.75	0.38	0.51
10	Chlorambucil (Celkeran, Chloramax)	2mg	tabs	LPG	2.15	0.75	0.33	0.44
11	Cisplatin (Cistero-10, Abiplatin, Kemoplat)	10mg	vial	LPG	17.10	5.03	2.61	0.52
12	Cisplatin (Cistero-50, Kemoplat, Celplat)	50mg	vial	LPG	15.53	7.25	2.37	0.33
13	Cyclophosphamide (Cyphos)	1g	vial	LPG	9.58	8.27	1.46	0.18
14	Cyclophosphamide (Endoxan, Cytoxan)	50mg	tabs	OB	0.50	0.30	0.08	0.25

15	Cyclophosphamide (Cycloxan, Phoxelon)	50mg	tabs	LPG	3.30	0.30	0.50	1.66
16	Cyclophosphamide (Phoxelon-500, Cyphos)	500mg	vial	LPG	3.55	5.24	0.54	0.10
17	Cytarabin (Cytalon-100)	100mg	vial	LPG	13.63	3.48	2.08	0.60
18	Dacarbazine (Celdaz, Dacarex)	200mg	vial	LPG	17.18	6.81	2.62	0.39
19	Dactinomycin/ Actinomysin D (Dacilon)	0.5mg	vial	LPG	17.35	8.70	2.65	0.30
20	Daunorubicin (Daunotec)	20mg	vial	LPG	39.24	19.32	5.99	0.31
21	Docetaxel Trihydrate (Docetero-20)	20mg	vial	LPG	44.27	40.50	6.76	0.17
22	Docetaxel Trihydrate (Daxotel, Docetero-80, Docetaxel Sandoz)	80mg	vial	LPG	109.53	47.97	16.72	0.35
23	Doxorubicin HCL	10mg	vial	LPG	4.63	2.12	0.71	0.33
24	Doxorubicin HCL (Doxinyl -50, Doxorubicine HCl Sandoz)	50mg	vial	LPG	16.35	5.41	2.50	0.46
25	Epirubicin (Epiget-50, Epiruba)	50mg	vial	LPG	49.56	21.68	7.57	0.35
26	Etoposide (Posid, Etopa, Etovel, Oncosid-100)	100mg	vial	LPG	7.19	2.02	1.10	0.54
27	Exemestane (Aromasin)	25mg	tabs	OB	5.27	2.09	0.81	0.39
28	Filgrastim (Neupogen, Zarzio, Nivestim, Accofil)	300mcg	vial	OB	57.82	75.57	8.83	0.12
29	Fluorouracil (Raciwel)	500mg	vial	LPG	2.31	0.26	0.35	1.35

30	Gemcitabine (Gemget-1000, Gemwel)	1000mg	vial	LPG	113.99	25.27	17.41	0.69
31	Ifosfamide + Mesna	1g	vial	LPG	9.91	26.71	1.51	0.06
32	Imatinib (Veenat-100)	100mg	tabs	LPG	2.64	0.69	0.40	0.58
33	Imatinib	400mg	tabs	LPG	19.82	25.21	3.03	0.12
34	L-Asparaginase (Bionase)	10,000iu	vial	LPG	57.82	52.88	8.83	0.17
35	Melphalan (Alkacel-2)	2mg	tabs	LPG	3.30	0.9889	0.50	0.51
36	Mercaptopurine	50mg	tabs	LPG	0.38	2.24	0.06	0.03
37	Methotrexate (Biotrexate)	2.5mg	tabs	LPG	0.25	0.06	0.04	0.60
38	Oxaliplatin	100mg	vial	LPG	69.38	74.77	10.59	0.14
39	Paclitaxel (Intaxel, Ataxil, Paclitec-100, Pacliwel, Paclitec-100, Paclitaxel Sandoz)	100mg	vial	LPG	33.87	11.08	5.17	0.47
40	Tamoxifen (Tamoxifen-Teva)	10mg	tabs	LPG	0.64	0.08	0.10	1.23
41	Tamoxifen (Cytotam)	20mg	tabs	LPG	0.56	0.12	0.09	0.69
42	Thalidomide (Thalix-100)	100mg	cap	LPG	2.18	1.31	0.33	0.25
43	Vinblastine (Chemoblast)	10mg	vial	LPG	12.39	4.98	1.89	0.38
44	Vincristine (Biocristine-AQ, Vincristine Medcrist, Vinlon-1, Vincristine Micristin, Cytocristin)	1mg	vial	LPG	5.78	2.54	0.88	0.35
45	Vinorelbine (Vinelbine)	50mg	vial	LPG	115.64	29.01	17.66	0.61
46	Zoledronic Acid (Zoldron, Zelodro-Denk)	4mg/5ml	vial	LPG	46.92	23.45	7.16	0.31
