# Supplementary material for: A comprehensive survey of cancer medicines prices, availability and affordability in Ghana
Source: PLoS One. 2023 May 3;18(5):e0279817. doi: 10.1371/journal.pone.0279817 (PMC10155977; doi:10.1371/journal.pone.0279817)
Supplement: S8 Table — (PDF) [file pone.0279817.s008.pdf]

S8 Table 8a. Affordability of Cancer Medicines in Public Hospitals

Medicine Name	Medicine Strength	Dosage Form	Target Pack Size	Medicine Type	Median Price (USD)	Dosage (based on an 80kg adult)	Treatment per Month (Number of Vials/Tabs)	Treatment Cost per Month (USD)	Daily Wage (USD)	Affordability
Bicalutamide	50mg	tabs	1	OB	1.43	1 tab/day	30	42.77	2.07	21
Bicalutamide	50mg	tabs	1	LPG	0.48	1 tab/day	30	14.27	2.07	7
Bicalutamide	150mg	tabs	1	OB	51.97	1 tab/day	30	1558.98	2.07	753
Bicalutamide	150mg	tabs	1	LPG	0.53	1 tab daily	30	15.81	2.07	8
Bleomycin	15 IU PFR	vial	1	LPG	18.17	15000 IU/2x weekly	8	145.38	2.07	70
Carboplatin	150mg	vial	1	LPG	26.76	400mg/m2/ month	3	80.29	2.07	39
Carboplatin	450mg	vial	1	LPG	68.89	400mg/m2/ month	1	68.89	2.07	33
Chlorambucil	2mg	tabs	1	LPG	1.73	0.2 mg/kg/day	240	416.30	2.07	201
Cisplatin	10mg	vial	1	LPG	11.73	120 mg/ m2/month	12	140.75	2.07	68
Cisplatin	50mg	vial	1	LPG	11.89	120 mg/ m2/month	3	35.68	2.07	17
Cyclophosphamide	1g	vial	1	LPG	5.95	300 mg/m2 /day	9	53.52	2.07	26
Cyclophosphamide	500mg	vial	1	LPG	2.97	300 mg/ m2/day	18	53.52	2.07	26
Dacarbazine	200mg	vial	1	LPG	14.04	250 mg/ m2/day	13	182.55	2.07	88

						for 10 days (monthly)				
Docetaxel Trihydrate	20mg	vial	1	LPG	33.04	75mg/m2/month	4	132.16	2.07	64
Docetaxel Trihydrate	80mg	vial	1	LPG	84.25	75mg/m2/month	1	84.25	2.07	41
Docetaxel Trihydrate	120mg	vial	1	LPG	90.86	75mg/m2/month	1	90.86	2.07	44
Doxorubicin HCL	50mg	vial	1	LPG	16.19	75mg/m2/month	2	32.38	2.07	16
Epirubicin	50mg	vial	1	LPG	46.26	90 mg/m² x2/month	4	185.02	2.07	89
Etoposide	100mg	vial	1	LPG	6.28	100 mg/m2 /day x 5 days (monthly)	5	31.39	2.07	15
Filgrastim	300mcg	vial	1	OB	3.63	5 µg/kg /day	40	145.38	2.07	70
Fluorouracil	50mg	vial	1	LPG	1.16	15mg/kg/week	100	115.64	2.07	56
Fluorouracil	500mg	vial	1	LPG	2.15	15mg/kg/week	10	21.48	2.07	10
Gemcitabine	1000mg	vial	1	LPG	90.86	1000 mg/m2 /week	4	363.44	2.07	176
Goserelin	3.6mg	vial	1	OB	104.56	3.6mg inj/ month	1	104.56	2.07	51

Goserelin	10.8mg	vial	1	OB	256.19	10.8mg inj/ every 3 months	1	256.19	2.07	124
Ifosfamide + Mesna inj	1g	vial	1	OB	9.09	10 g/m²/ month	10	90.86	2.07	44
Irinotecan	100mg	vial	1	LPG	72.69	350mg/m2/ month	4	290.75	2.07	140
Leuprolide Acetate	11.25mg	vial	1	LPG	132.16	11.25 mg/ month	1	132.16	2.07	64
Liposomal Doxorubicin	20mg	vial	1	OB	4.96	50 mg/m2 /month	1	4.96	2.07	2
Liposomal Doxorubicin	20mg	vial	1	LPG	13.22	50 mg/m2 /month	1	13.22	2.07	6
Liposomal Doxorubicin	50mg	vial	1	OB	3.30	50 mg/m2 /month	1	3.30	2.07	2
Liposomal Doxorubicin	50mg	vial	1	LPG	14.04	50 mg/m2 /month	1	14.04	2.07	7
Methotrexate	50mg	vial	1	LPG	5.70	25 mg / week	2	11.40	2.07	6
Oxaliplatin	100mg	vial	1	LPG	66.08	85mg/m2/2x monthly	2	132.16	2.07	64
Paclitaxel	100mg	vial	1	LPG	33.54	260mg/m2/every 3 weeks (monthly)	6	201.21	2.07	97
Tamoxifen	20mg	tabs	1	LPG	8.26	20mg/day	30	247.80	2.07	120
Trastuzumab	600mg	vial	1	OB	646.59	600 mg/ every 3	2	1293.19	2.07	625

						weeks (monthly)				
Vincristine	1mg	vial	1	LPG	2.89	2 mg/ week	8	23.13	2.07	11
Vinorelbine	50mg	vial	1	LPG	74.34	25mg/m²/week	2	148.68	2.07	72
Zoledronic Acid	4mg/5ml	vial	1	LPG	38.00	4 mg/ month	1	38.00	2.07	18
