# Supplementary material for: A comprehensive survey of cancer medicines prices, availability and affordability in Ghana
Source: PLoS One. 2023 May 3;18(5):e0279817. doi: 10.1371/journal.pone.0279817 (PMC10155977; doi:10.1371/journal.pone.0279817)
Supplement: S9 Table — (PDF) [file pone.0279817.s009.pdf]

S9 Table 8b. Affordability of Cancer Medicines in Private Hospitals

Medicine Name	Medicine Strength	Dosage Form	Target Pack size	Medicine Type	Median Price (USD)	Dosage (based on an 80kg adult)	Treatment per Month (Number of Vials/Tabs)	Treatment Cost per Month (USD)	Daily Wage (USD)	Affordability
Abiraterone	250mg	tabs	1	OB	6.88	1000mg /day	120.00	826.00	2.07	399
Anastrozole	1mg	tabs	1	OB	0.71	1 tab/day	30.00	21.24	2.07	10
Bevacizumab	400mg	vial	1	OB	1321.60	1600 mg/month	4.00	5286.40	2.07	2554
Bleomycin	15 IU PFR	vial	1	LPG	46.26	15000 IU/ 2x weekly	8.00	370.05	2.07	179
Capecitabine	500mg	tabs	1	OB	2.62	2500mg/ m2/day (21 days cycle)	105.00	274.64	2.07	133
Carboplatin	450mg	vial	1	LPG	99.12	400mg/m2/ month	1.00	99.12	2.07	48
Cisplatin	50mg	vial	1	LPG	15.69	120 mg/m2 /month	3.00	47.08	2.07	23
Cyclophosphamide	1g	vial	1	LPG	7.85	300 mg/m2 /day	9.00	70.62	2.07	34
Cyclophosphamide	50mg	tabs	1	OB	0.41	300mg/day	180.00	74.34	2.07	36
Docetaxel Trihydrate	80mg	vial	1	LPG	132.57	75mg/m2/ month	1.00	132.57	2.07	64
Doxorubicin HCL	50mg	vial	1	LPG	14.12	75mg/m2/ month	2.00	28.25	2.07	14

Epirubicin	50mg	vial	1	OB	85.90	90 mg/m² x2/month	4.00	343.62	2.07	166
Etoposide	100mg	vial	1	LPG	16.52	100 mg/m2 /day x 5 days (monthly)	5.00	82.60	2.07	40
Fluorouracil	500mg	vial	1	LPG	4.13	15mg/kg /week	10.00	41.30	2.07	20
Gemcitabine	1000mg	vial	1	LPG	165.20	1000 mg/ m2/week	4.00	660.80	2.07	319
Oxaliplatin	100mg	vial	1	LPG	132.16	85mg/m2 /2x monthly	2.00	264.32	2.07	128
Paclitaxel	100mg	vial	1	LPG	49.56	260mg/m2/ 3 weeks (monthly)	6.00	297.36	2.07	144
Sorafenib	200mg	tabs	1	LPG	7.16	400mg/2x day	120.00	859.04	2.07	415
Trastuzumab	600mg	vial	1	OB	1321.60	600 mg/3 weeks (monthly)	2.00	2643.20	2.07	1277
Vincristine	1mg	vial	1	LPG	2.97	2 mg/week	8.00	23.79	2.07	11
Zoledronic Acid	4mg/5ml	vial	1	LPG	82.60	4 mg/ month	1.00	82.60	2.07	40
