# Supplementary material for: A comprehensive survey of cancer medicines prices, availability and affordability in Ghana
Source: PLoS One. 2023 May 3;18(5):e0279817. doi: 10.1371/journal.pone.0279817 (PMC10155977; doi:10.1371/journal.pone.0279817)
Supplement: S10 Table — (PDF) [file pone.0279817.s010.pdf]

**S10 Table 8c.** Affordability of Cancer Medicines in Private Pharmacies

| Medicine Name | Medicine Strength | Dosage Form | Target pack size | Medicine Type | Median price (USD) | Dosage (based on an 80kg adult) | Treatment per month (Number of Vials/Tabs) | Treatment Cost per month (USD) | Daily Wage (USD) | Affordability |
|---|---|---|---|---|---|---|---|---|---|---|
| Abiraterone | 250mg | tabs | 1 | OB | 5.49 | 1000mg /day | 120 | 659.15 | 2.07 | 318 |
| Anastrozole | 1mg | tabs | 1 | OB | 1.14 | 1 tab/day | 30 | 34.05 | 2.07 | 16 |
| Anastrozole | 1mg | tabs | 1 | LPG | 0.50 | 2 tab/day | 31 | 15.36 | 2.07 | 7 |
| Bevacizumab | 400mg | vial | 1 | OB | 307.44 | 1600 mg/ month | 4 | 1229.75 | 2.07 | 594 |
| Bicalutamide | 50mg | tabs | 1 | OB | 1.58 | 1 tab/day | 30 | 47.44 | 2.07 | 23 |
| Bicalutamide | 150mg | tabs | 1 | OB | 3.47 | 2 tab/day | 31 | 107.55 | 2.07 | 52 |
| Bleomycin | 15 IU PFR | vial | 1 | LPG | 26.43 | 15000 IU/ 2x week | 8 | 211.46 | 2.07 | 102 |
| Bortezomib | 3.5mg | vial | 1 | LPG | 227.98 | 1.3mg/m2 twice a week for a 3-week cycle | 3 | 683.93 | 2.07 | 330 |
| Capecitabine | 500mg | tabs | 1 | OB | 2.64 | 2500mg/m2 daily for a 21 day cycle | 105 | 277.54 | 2.07 | 134 |
| Carboplatin | 150mg | vial | 1 | LPG | 27.84 | 400mg/m2/ month | 3 | 83.51 | 2.07 | 40 |

| | | | | | | | | | | |
|---|---|---|---|---|---|---|---|---|---|---|
| Carboplatin | 450mg | vial | 1 | LPG | 74.34 | 400mg/m2/ month | 1 | 74.34 | 2.07 | 36 |
| Chlorambucil | 2mg | tabs | 1 | OB | 2.51 | 0.2 mg/ kg/day | 240 | 602.65 | 2.07 | 291 |
| Chlorambucil | 2mg | tabs | 1 | LPG | 2.15 | 0.2 mg/ kg/day | 241 | 517.57 | 2.07 | 250 |
| Cisplatin | 10mg | vial | 1 | LPG | 17.10 | 120 mg/ m2/month | 12 | 205.18 | 2.07 | 99 |
| Cisplatin | 50mg | vial | 1 | LPG | 15.53 | 120 mg/ m2/month | 3 | 46.59 | 2.07 | 23 |
| Cyclophospha mide | 1g | vial | 1 | LPG | 9.58 | 300 mg/ m2/day | 9 | 86.23 | 2.07 | 42 |
| Cyclophospha mide | 50mg | tabs | 1 | OB | 0.50 | 300mg/day | 180 | 89.21 | 2.07 | 43 |
| Cyclophospha mide | 50mg | tabs | 1 | LPG | 3.30 | 300mg/ day | 181 | 598.02 | 2.07 | 289 |
| Cyclophospha mide | 500mg | vial | 1 | LPG | 3.55 | 300 mg/ m2/day | 18 | 63.93 | 2.07 | 31 |
| Cytarabin | 100mg | vial | 1 | LPG | 13.63 | 2 mg/kg/ day for 10 days, then increase to 4mg/kg/day | 80 | 1090.32 | 2.07 | 527 |
| Dacarbazine | 200mg | vial | 1 | LPG | 17.18 | 250 mg/m2/day for 10 days (monthly) | 13 | 223.35 | 2.07 | 108 |

| | | | | | | | | | | |
|---|---|---|---|---|---|---|---|---|---|---|
| Dacarbazine | 500mg | vial | 1 | LPG | 17.35 | 250mg/m2/day for 10 days (monthly) | 5 | 86.73 | 2.07 | 42 |
| Dactinomycin/ Actinomysin D | 0.5mg | vial | 1 | LPG | 17.35 | 0.6mg/m2/day for 10 days (monthly) | 12 | 208.15 | 2.07 | 101 |
| Daunorubicin | 20mg | vial | 1 | LPG | 39.24 | 60 mg/m2 on alternate days x 3 (monthly) | 9 | 353.12 | 2.07 | 171 |
| Docetaxel Trihydrate | 20mg | vial | 1 | LPG | 44.27 | 75mg/m2/ month | 4 | 177.09 | 2.07 | 86 |
| Docetaxel Trihydrate | 80mg | vial | 1 | LPG | 109.53 | 75mg/m2 /month | 1 | 109.53 | 2.07 | 53 |
| Doxorubicin HCL | 10mg | vial | 1 | LPG | 4.63 | 75mg/m2/ month | 8 | 37.00 | 2.07 | 18 |
| Doxorubicin HCL | 50mg | vial | 1 | LPG | 16.35 | 75mg/m2/ month | 2 | 32.71 | 2.07 | 16 |
| Epirubicin | 50mg | vial | 1 | LPG | 49.56 | 90 mg/m² x2 (monthly) | 4 | 198.24 | 2.07 | 96 |
| Etoposide | 100mg | vial | 1 | LPG | 7.19 | 100 mg/m2/ day x 5 days (monthly) | 5 | 35.93 | 2.07 | 17 |
| Exemestane | 25mg | tabs | 1 | OB | 5.27 | 1 tab/day | 30 | 158.20 | 2.07 | 76 |
| Filgrastim | 300mcg | vial | 1 | OB | 57.82 | 5 µg/kg/day | 40 | 2312.80 | 2.07 | 1117 |

| | | | | | | | | | | |
|---|---|---|---|---|---|---|---|---|---|---|
| Fluorouracil | 500mg | vial | 1 | LPG | 2.31 | 15mg/kg/ week | 10 | 23.13 | 2.07 | 11 |
| Gemcitabine | 1000mg | vial | 1 | LPG | 113.99 | 1000 mg/m2 /week | 4 | 455.95 | 2.07 | 220 |
| Goserelin | 3.6mg | vial | 1 | OB | 117.90 | 3.6mg inj/month | 1 | 117.90 | 2.07 | 57 |
| Goserelin | 10.8mg | vial | 1 | OB | 295.72 | 10.8mg inj/every 3 monthly | 1 | 295.72 | 2.07 | 143 |
| Hydreaxyurea | 250mg | tabs | 1 | OB | 0.62 | 30 mg/ kg/day | 288 | 178.42 | 2.07 | 86 |
| Ifosfamide + Mesna inj | 1g | vial | 1 | LPG | 9.91 | 10 g/m² /month | 10 | 99.12 | 2.07 | 48 |
| Imatinib | 100mg | tabs | 1 | LPG | 2.64 | 400 mg/day | 120 | 317.18 | 2.07 | 153 |
| Imatinib | 400mg | tabs | 1 | LPG | 19.82 | 800 mg/day | 60 | 1189.44 | 2.07 | 575 |
| L-Asparaginase | 10,000iu | vial | 1 | LPG | 57.82 | 5000 U/m2 /every 3 days | 5 | 289.10 | 2.07 | 140 |
| Lenalidomide | 10mg | capsules | 1 | LPG | 6.61 | 25 mg/day/for 3 weeks cycle | 53 | 350.22 | 2.07 | 169 |
| Leuprolide | 3.75mg | vial | 1 | OB | 235.24 | 3.75 mg/month | 1 | 235.24 | 2.07 | 114 |
| Leuprolide Acetate | 11.25mg | vial | 1 | LPG | 177.84 | 11.25 mg/month | 1 | 177.84 | 2.07 | 86 |
| Melphalan | 2mg | tabs | 1 | LPG | 3.30 | 0.2 mg/kg | 40 | 132.16 | 2.07 | 64 |

| | | | | | | | | | | |
|---|---|---|---|---|---|---|---|---|---|---|
| | | | | | | /5 days (monthly) | | | | |
| Mercaptopurine | 50mg | tabs | 1 | LPG | 0.38 | 2.5 mg/kg /day | 120 | 45.60 | 2.07 | 22 |
| Mercaptopurine | 150mg | tabs | 1 | OB | 5.02 | 2.5 mg/kg /day | 40 | 200.88 | 2.07 | 97 |
| Methotrexate | 2.5mg | tabs | 1 | LPG | 0.25 | 7.5 mg / week | 12 | 2.97 | 2.07 | 1 |
| Methotrexate | 50mg | vial | 1 | LPG | 7.52 | 25 mg / week | 2 | 15.03 | 2.07 | 7 |
| Mitomycin | 10mg | vial | 1 | LPG | 37.67 | 10mg/m2 /month | 1 | 37.67 | 2.07 | 18 |
| Oxaliplatin | 100mg | vial | 1 | LPG | 69.38 | 85mg/m2/ 2x month | 2 | 138.77 | 2.07 | 67 |
| Paclitaxel | 100mg | vial | 1 | LPG | 33.87 | 260mg/m2 every 3 weeks (monthly) | 6 | 203.20 | 2.07 | 98 |
| Sorafenib | 200mg | tabs | 1 | LPG | 2.48 | 400mg/2x day | 120 | 297.36 | 2.07 | 144 |
| Tamoxifen | 10mg | tabs | 1 | LPG | 0.64 | 20mg/day | 60 | 38.66 | 2.07 | 19 |
| Tamoxifen | 20mg | tabs | 1 | LPG | 0.56 | 20mg/day | 30 | 16.85 | 2.07 | 8 |
| Thalidomide | 50mg | caps | 1 | LPG | 28.33 | 200mg/day | 120 | 3399.82 | 2.07 | 1642 |
| Thalidomide | 100mg | cap | 1 | LPG | 2.18 | 200mg/day | 60 | 130.69 | 2.07 | 63 |
| Trastuzumab | 600mg | vial | 1 | OB | 581.21 | 600 mg/ | 2 | 1162.43 | 2.07 | 562 |

| | | | | | | | | | | |
|---|---|---|---|---|---|---|---|---|---|---|
| | | | | | | every 3 weeks (monthly) | | | | |
| Triptorelin Acetate | 3.75mg | vial | 1 | OB | 107.38 | 3.75mg/ month | 1 | 107.38 | 2.07 | 52 |
| Vinblastine | 10mg | vial | 1 | LPG | 12.39 | 6 mg/m2/ week | 3 | 37.17 | 2.07 | 18 |
| Vincristine | 1mg | vial | 1 | LPG | 5.78 | 2 mg/week | 8 | 46.26 | 2.07 | 22 |
| Vinorelbine | 50mg | vial | 1 | LPG | 115.64 | 25mg/m²/ week | 2 | 231.28 | 2.07 | 112 |
| Zoledronic Acid | 4mg/5ml | vial | 1 | LPG | 46.92 | 4 mg/month | 1 | 46.92 | 2.07 | 23 |
