# Supplementary material for: A comprehensive survey of cancer medicines prices, availability and affordability in Ghana
Source: PLoS One. 2023 May 3;18(5):e0279817. doi: 10.1371/journal.pone.0279817 (PMC10155977; doi:10.1371/journal.pone.0279817)

PLOS ONE

A Comprehensive Survey of Cancer Medicines Pricing, Availability and Affordability in Ghana

--Manuscript Draft--

**Manuscript Number:**	PONE-D-21-38310
**Article Type:**	Research Article
**Full Title:**	A Comprehensive Survey of Cancer Medicines Pricing, Availability and Affordability in Ghana
**Short Title:**	Cancer Medicines Pricing, Availability and Affordability in Ghana
**Corresponding Author:**	Phyllis Benonia Ocran Mattila, MPH<br>University of Huddersfield<br>Huddersfield, UNITED KINGDOM
**Keywords:**	Cancer, Medicine, Pricing, Availability, Affordability, Access, Ghana, LMICs, Africa.
**Abstract:**	In Ghana (Gh), cancer prices are characterized by high retail markups, forex fluctuations and high variation in prices of medicines. Most patients cannot afford the cancer medicines. There is a problem of unaffordability and limited availability of essential cancer medicines which suggests potential inequity in patient access to cancer medicines. Prices of cancer medicines are a major contributor to the cost of treatment for cancer patients and the comparison of these cost was assessed to determine the affordability. The objective was to assess the prices, availability, and affordability of cancer medicines in Ghana. An adapted World Health Organization (WHO)/ Health Action International (HAI) methodology was used for measuring medicine prices. The availability of cancer medicines was assessed as percentage of health facilities stocked with listed medicines. The Price U.S dollars (USD) variation between the same medicine's highest- and lowest-priced product, Originator Brand (OB) and its Lowest Priced Generic (LPG) was compared. Median Unit Price (MUP) was compared with the International Reference Price (IRP) to obtain a Median Price Ratio (MPR). The affordability of cancer medicines based on treatment affordability in relation to the daily wage of the unskilled Lowest-Paid Government Worker (LPGW) was also determined. The median prices (USD) of LPG ranged as (0.25 – 227.98), and OB (0.41-1321.60). The adjusted MPRs of OBs and LPGs ranged from 0.01 -10.15. Some variations between highest- and lowest-priced were 20.60 times more expensive. Only two cancer conditions are covered by the National Health Insurance Scheme (NHIS). Affordability calculations showed that patients with multiple myeloma cancer would need 1642 days wages to afford treatment. Availability was very low, with a median availability of 10.3%, ranging from 3.4% (lowest) to 48.3% (highest), which is far below the WHO 80% target. The availability of cancer medicines was very low, and affordability remains suboptimal. There was a considerable variation in the price of different brands of cancer medicines available in Ghana.
**Order of Authors:**	Phyllis Benonia Ocran Mattila, MPH
	Richard Berko Britwum, PHD
	Zaheer Ud-Din Babar, PHD
**Additional Information:**	
**Question**	**Response**
**Financial Disclosure**<br><br>Enter a financial disclosure statement that describes the sources of funding for the work included in this submission. Review the submission guidelines for detailed requirements. View published research articles from *PLOS ONE* for specific	The author(s) received no specific funding for this work.

Powered by Editorial Manager® and ProduXion Manager® from Aries Systems Corporation

examples.<br><br>This statement is required for submission and **will appear in the published article** if the submission is accepted. Please make sure it is accurate.<br><br>**Unfunded studies**<br>Enter: *The author(s) received no specific funding for this work.*<br><br>**Funded studies**<br>Enter a statement with the following details:<br>• Initials of the authors who received each award<br>• Grant numbers awarded to each author<br>• The full name of each funder<br>• URL of each funder website<br>• Did the sponsors or funders play any role in the study design, data collection and analysis, decision to publish, or preparation of the manuscript?<br>• **NO** - Include this sentence at the end of your statement: *The funders had no role in study design, data collection and analysis, decision to publish, or preparation of the manuscript.*<br>• **YES** - Specify the role(s) played.<br><br>* typeset	
**Competing Interests**<br><br>Use the instructions below to enter a competing interest statement for this submission. On behalf of all authors, disclose any competing interests that could be perceived to bias this work—acknowledging all financial support and any other relevant financial or non-financial competing interests.<br><br>This statement is required for submission and **will appear in the published article** if the submission is accepted. Please make sure it is accurate and that any funding sources listed in your Funding Information later in the submission form are also declared in your Financial Disclosure statement.	The authors have declared that no competing interests exist.

Powered by Editorial Manager® and ProduXion Manager® from Aries Systems Corporation

View published research articles from *PLOS ONE* for specific examples.<br><br>**NO authors have competing interests**<br><br>Enter: *The authors have declared that no competing interests exist.*<br><br>**Authors with competing interests**<br><br>Enter competing interest details beginning with this statement:<br><br>*I have read the journal's policy and the authors of this manuscript have the following competing interests: [insert competing interests here]*<br><br>* typeset	
**Ethics Statement**<br><br>Enter an ethics statement for this submission. This statement is required if the study involved:<br><br>• Human participants<br>• Human specimens or tissue<br>• Vertebrate animals or cephalopods<br>• Vertebrate embryos or tissues<br>• Field research<br><br>Write "N/A" if the submission does not require an ethics statement.<br><br>General guidance is provided below. Consult the submission guidelines for detailed instructions. **Make sure that all information entered here is included in the Methods section of the manuscript.**	N/A- this study did not require human subjects. It collected data on medicine prices only. It did not involve minors or participants. Ethical clearance to collect the data in the health facilities was granted by the Ethical committees of the University of Huddersfield, KBTH, KATH and Ghana Health Service Research Committees respectively.

**Format for specific study types**<br><br>**Human Subject Research (involving human participants and/or tissue)**<br>• Give the name of the institutional review board or ethics committee that approved the study<br>• Include the approval number and/or a statement indicating approval of this research<br>• Indicate the form of consent obtained (written/oral) or the reason that consent was not obtained (e.g. the data were analyzed anonymously)<br><br>**Animal Research (involving vertebrate animals, embryos or tissues)**<br>• Provide the name of the Institutional Animal Care and Use Committee (IACUC) or other relevant ethics board that reviewed the study protocol, and indicate whether they approved this research or granted a formal waiver of ethical approval<br>• Include an approval number if one was obtained<br>• If the study involved *non-human primates*, add *additional details* about animal welfare and steps taken to ameliorate suffering<br>• If anesthesia, euthanasia, or any kind of animal sacrifice is part of the study, include briefly which substances and/or methods were applied<br><br>**Field Research**<br><br>Include the following details if this study involves the collection of plant, animal, or other materials from a natural setting:<br>• Field permit number<br>• Name of the institution or relevant body that granted permission	
**Data Availability**<br><br>Authors are required to make all data underlying the findings described fully available, without restriction, and from the time of publication. PLOS allows rare exceptions to address legal and ethical concerns. See the PLOS Data Policy and FAQ for detailed information.	Yes - all data are fully available without restriction

A Data Availability Statement describing where the data can be found is required at submission. Your answers to this question constitute the Data Availability Statement and **will be published in the article**, if accepted.<br><br>**Important:** Stating 'data available on request from the author' is not sufficient. If your data are only available upon request, select 'No' for the first question and explain your exceptional situation in the text box.<br><br>Do the authors confirm that all data underlying the findings described in their manuscript are fully available without restriction?	
**Describe where the data may be found in full sentences. If you are copying our sample text, replace any instances of XXX with the appropriate details.**<br><br>• If the data are **held or will be held in a public repository**, include URLs, accession numbers or DOIs. If this information will only be available after acceptance, indicate this by ticking the box below. For example: *All XXX files are available from the XXX database (accession number(s) XXX, XXX.).*<br>• If the data are all contained **within the manuscript and/or Supporting Information files**, enter the following: *All relevant data are within the manuscript and its Supporting Information files.*<br>• If neither of these applies but you are able to provide **details of access elsewhere**, with or without limitations, please do so. For example:<br><br>*Data cannot be shared publicly because of [XXX]. Data are available from the XXX Institutional Data Access / Ethics Committee (contact via XXX) for researchers who meet the criteria for access to confidential data.*<br><br>*The data underlying the results presented in the study are available from (include the name of the third party*	All relevant data are within the manuscript and its Supporting Information files.

Powered by Editorial Manager® and ProduXion Manager® from Aries Systems Corporation

and contact information or URL).<br>• This text is appropriate if the data are owned by a third party and authors do not have permission to share the data.<br><br>* typeset	
Additional data availability information:	Tick here if your circumstances are not covered by the questions above and you need the journal’s help to make your data available.

Powered by Editorial Manager® and ProduXion Manager® from Aries Systems Corporation

Manuscript

Click here to access/download;Manuscript;211203 Pricing Availability Affordability Cancer Meds Ghana.docx

Title: A Comprehensive Survey of Cancer Medicines Pricing, Availability and Affordability in Ghana

Authors: Phyllis Ocran Mattila [1], Richard Berko Biritwum[2], Zaheer-Ud-Din Babar [3].

1. Postgraduate Research Student, Department of Pharmacy, University of Huddersfield, Queensgate, HD1 3DH, Huddersfield, United Kingdom, Tel: +265-9982-97090, Email: phyllis.ocran@hud.ac.uk
2. Professor of Public Health, School of Public Health, Department of Community Health, University of Ghana Medical School. Accra, Ghana. Tel: +233-2432-36839 e-mail; biritwum@africaonline.com.gh
3. Professor in Medicines and Healthcare, Department of Pharmacy, University of Huddersfield, Queensgate, HD1 3DH, Huddersfield, United Kingdom, Tel (office): +44 1484471471. Email: z.babar@hud.ac.uk

Corresponding Author: Phyllis Ocran Mattila

Postgraduate Research Student, Department of Pharmacy, University of Huddersfield, Queensgate, HD1 3DH, Huddersfield, United Kingdom.

Tel: +265-9982-97090/+45-71362625

Email: phyllis.ocran@hud.ac.uk

Abstract

In Ghana (Gh), cancer prices are characterized by high retail markups, forex fluctuations and high variation in prices of medicines. Most patients cannot afford the cancer medicines. There is a problem of unaffordability and limited availability of essential cancer medicines which suggests potential inequity in patient access to cancer medicines. Prices of cancer medicines are a major contributor to the cost of treatment for cancer patients and the comparison of these cost was assessed to determine the affordability. The objective was to assess the prices, availability, and affordability of cancer medicines in Ghana. An adapted World Health Organization (WHO)/ Health Action International (HAI) methodology was used for measuring medicine prices. The availability of cancer medicines was assessed as percentage of health facilities stocked with listed medicines. The Price U.S dollars (USD) variation between the same medicine's highest- and lowest-priced product, Originator Brand (OB) and its Lowest Priced Generic (LPG) was compared. Median Unit Price (MUP) was compared with the International Reference Price (IRP) to obtain a Median Price Ratio (MPR). The affordability of cancer medicines based on treatment affordability in relation to the daily wage of the unskilled Lowest-Paid Government Worker (LPGW) was also determined. The median prices (USD) of LPG ranged as (0.25 – 227.98), and OB (0.41-1321.60). The adjusted MPRs of OBs and LPGs ranged from 0.01 -10.15. Some variations between highest- and lowest-priced were 20.60 times more expensive. Only two cancer conditions are covered by the National Health Insurance Scheme (NHIS). Affordability calculations showed that patients with multiple myeloma cancer would need 1642 days wages to afford treatment. Availability was very low, with a median availability of 10.3%, ranging from 3.4% (lowest) to 48.3% (highest), which is far below the WHO 80% target. The availability of

cancer medicines was very low, and affordability remains suboptimal. There was a considerable variation in the price of different brands of cancer medicines available in Ghana.

Introduction

Cancer is a great public health issue. The estimated global cancer burden is 19.3 million new cases and 9.9 million deaths in 2020. This may increase to 30.2 million new cases by 2040 [1]. In Africa, new cancer cases were 1.1 million in 2020 and is projected to reach 2.1 in 2040 [1]. In 2020, Ghana had 24,009 cancer cases (14,078 in females, 9,931 in males) and 15,089 deaths, with new cases projected to be 44,475 (25,518 in females, 18,957 in males) in 2040 [1].The most common cancers in adults are; breast (18.7%), liver (14.4%), cervix uterus (11.6%), prostate (8.9%), Non-Hodgkin's lymphoma (5%), ovary (4.2%), Colo rectum (3.3%), and other cancers (33.9%). And for children; Non-Hodgkin's lymphoma (20%), leukemia (16.1%), kidney (11%), BNS (7.2%), liver (2.2%), Hodgkin's lymphoma (1.9%), naso pharynx (1.7%) and other cancers (40%) [1].

This has serious economic implications. The cancer burden and mortality can be reduced with early diagnosis and treatment [2]. This depends on the equitable access to available and affordable cancer medicines. In low- and middle-income countries (LMICs) improving health system strengthening is the key and it can improve various facets of medicine chain including access, affordability, and quality of medicines [3].

Over 20% of African countries have no access to cancer medicines at all, while access is limited and sporadic in other countries [4]. There are concerns about the lack of adequate access to both new and off-patent cancer medicines, with soaring prices cited as a main contributory factor [5].

Many cancer patients cannot afford the cost of treatment, especially if they lack an adequate insurance coverage, government reimbursement or funding [6]. Even with an insurance scheme, patients often suffered financial hardships because of the prohibitive cost of cancer medicines [5]. In 2018, a study by WHO found that pricing of cancer medicines was disproportionately higher than other types of pharmaceuticals and therapies. Prices increased in the absence of regulations and non-uniform pricing policies, resulting in inefficient cost-shifting activities and potential inequity. Thus, a greater level of price control can lower prices and improve access [5]. In Ghana, cancer medicines are funded through health insurance or mainly through out of pocket payments popularly known as 'cash and carry'. Procurement of cancer medicines is done by private licensed medicine suppliers/wholesalers. All cancer medicines are imported, so prices are largely affected by forex fluctuations, as well as import tariffs and duties, which together with the profit markup cost, renders it very expensive and unaffordable to most patients [7]. With the minimum daily wage being approximately $2, the out of pocket cost of cancer care is very expensive and borne with much difficulty [8].

Currently, there are about 29 cancer facilities including government hospitals: Korle Teaching Hospital, Komfo Anokye Teaching Hospital, Cape Coast Teaching Hospital and Tamale Teaching Hospital and the 37 Military Specialist Hospital [9].

Most cancer patients do not have affordable access to treatment because of low availability of cancer medicines. The reasons include the absence of the cancer medicines on the Essential Medicines List (EML), inefficient supply chains, unreliable suppliers, budget capitation [5, 10]. Studies showed that in LMICs and in the 37 African countries subscribing to the WHO EML, the number of cancer medicines listed on the National Essential Medicines List (NEML) was eighteen and fifteen respectively, with many not listed [5, 11]. A WHO report showed that countries

especially Africa with lower national income had lower availability of cancer medicines, or availability only with higher out-of-pocket patient payments, especially for high-cost medicines, including targeted therapies [5]. Price transparency when enshrined in the National Medicines Policy (NMP), will help strengthen government's negotiating position and enhance their ability to obtain more affordable medicines [12]. The NMP, NEML and Ghana Standard Treatment Guidelines (STG), includes a pricing policy to improve pricing mechanisms and promote affordability of medicines [7,13,14]. The impact on affordability though is unclear. A major barrier to creating and implementing national childhood cancer strategies is a paucity of data on the cost of delivering childhood cancer medicines [15].

Several studies have examined the availability, price, and affordability of medicines [16], however none has been done to show the landscape in Ghana. This study, which to the best of our knowledge, is the first national comprehensive survey to be conducted in Ghana. It will add to scientific literature and improve the knowledge and equity considerations regarding pricing, affordability, availability, and access to cancer medicines. And thus, contribute to the development of pricing models, policies, and strategies to improve the survival and quality of life of the cancer patients. This study aims to assess the variation in prices of different brands of cancer medicines, its availability and affordability in the Public Hospitals, Private Hospitals, and Private Pharmacies of Ghana.

Methods

The study was conducted to examine the pricing, availability, affordability, and access to cancer medicines in Ghana, using an abridged WHO/HAI methodology [17]. A total of 29 facilities (7 public hospitals, 20 private pharmacies and 2 private hospitals) in 4 survey areas (Greater Accra, Ashanti, Central and Northern Regions respectively), were surveyed [9]. The 4 survey areas

denote a national survey, as they were the only available places with cancer medicines in Ghana at the time of the study. Each survey area covered a population of about 1.9 million to 5.9 million and reachable within one day's travel from Ghana 's main capital city, Accra [18]. (See Table 1).

Table 1. Number of Facilities (outlets sampled) and Geographical Location of Participating Cities [18].

**Region**	**Regional Population (2020) [19]**	**City Surveyed**	**Number of Facilities in each City**		
			**Public Hospital**	**Private Pharmacies**	**Private Hospitals**
Ashanti	5,924,498	Kumasi	1	7	0
Greater Accra	5,055,883	Accra	4	6	1
Central	2,605,492	Cape Coast	1	2	1
Northern	1,948,413	Tamale	1	5	0
Total Facilities			7	20	2

Three broad sectors; public (public hospitals), private (private pharmacies) and other (private hospitals) were analyzed for the survey.

Data was collected on Sixty-five (65) cancer medicines (with different strengths and dosage form). The selection was based on all that is currently available for use in the chemotherapy of neoplastic diseases in Ghana, and listed in the NEML, WHO EML and WHO Essential Medicines list for Children (EMLc) (See Table 2).

In this study, the term cancer medicines refer to cytotoxic and adjuvant medicines. The cytotoxic medicines include alkylating agents, antimetabolite analogs of folic acid, pyrimidine, and purine, natural products, hormones and hormone antagonists, and a variety of agents directed at specific molecular targets were assessed [19]. For each cancer medicine, data was collected on its availability, and on the low, high and median patient retail unit price (including if only one price

was found) of the OB, which is the original pharmaceutical product that was first authorized for marketing and the International Nonproprietary Name (INN)(generic name)/LPG, which is the lowest price found at each medicine outlet (place where medicines are dispensed to patients), of products other than the OB that contains the same active ingredient and marketed under a generic name [17]. Data was collected in local currency (Gh Cedis) and converted to USD using foreign exchange rate of (1 Gh Cedis = 0.1652 USD =), as at 06 October 2021[20].

For statistical analysis, the cancer medicines found in all 3 sectors were included. Each medicine's Median Unit Price (MUP) was calculated and then the 'mean-median' among the 3 sectors for matched sets of medicines was compared. The price, availability, and affordability were presented as means and standard deviations. The differences in prices, availability and affordability were calculated using Kruskal-Wallis test and $p < 0.05$ was used to indicate a significant difference.

An adapted WHO/HAI methodology questionnaire, with 3 sub sections on demographics, the medicine outlet and on the medicines availability and unit prices (price of pack divided by pack size i.e. the price per cap, tab, ml, dose, gram), was pilot tested and used for the data collection [17]. The data collection form is available from the authors upon request.

Data collection occurred between August 2020 to October 2020. The Data was double checked for completeness and accuracy with blanks and errors corrected and entered on a standardized computerized workbook and Microsoft excel spread sheet for analysis. Treatment regimens were taken from the Electronic Medicines Compendium (emc) [21], UK and the National Comprehensive Cancer Network (NCCN) treatment guidelines [22]. The 2015 Management Sciences Health (MSH), Supplier IRP were used to compare local cancer medicine prices to an international standard [23]. The MPR is the ratio of median local unit price of a surveyed medicine to its (IRP), and was calculated as follows:

MPR = Median local unit price (USD)/Median IRP (USD).

For this study, the MPR was calculated using prices having a deflation factor of 84.73% according to the Ghana Consumer Price Indices (CPI) for July 2014 to July 2019 [24], as the latest available IRPs were published in 2015 [23]. Like other studies, this price adjustment method was adopted to make the comparison between local and international prices more reliable [25, 26]. Normally, an MPR of one or less is taken as efficient procurement in the public sector, while below three, it is considered efficient for the private sector [27]. Procurement efficiency defined as the difference between the Highest-Priced Medicine (HPM) and Lowest-Priced Medicine (LPM) and Brand premiums between the OB and their LPG equivalents was determined [17]. The MUP was calculated rather than mean values. The percentage cost differential or price variation was calculated as; *Cost Differential (%) = [(Price of the Originator – Price of the generic)/Price of Originator] x 100.*

The Price Ratio (PR) between OB and LPG or HPM and LPM was calculated as*:*

Price ratio = Price of the OB/Price of the LPG or Price of the HPM/ Price of the LPM. If the calculated ratio is ≤1 the reported price is reasonable in the public sector. A value ≥3 in the private sector means Ghanaians pay more for the medicine than recommended by WHO [17].

The availability of the cancer medicines at the specified strengths for both OB and LPG will be analyzed by the percentage (%) of medicine outlets with cancer medicine(s) found on the day of data collection. Each medicine was reported as being listed or not listed on the NEML, WHO EML and EMLc. The medicines affordability was based on the WHO/HAI method by examining the costs of cancer medicines and comparing them with the daily wage of the LPGW [17]. The 2021 salary of the LPGW in Ghana is 12.53 Gh Cedis (2.07 USD) per day [7]. A month of cancer medicines was used to demonstrate the economic implication on a patient if they would have to

pay for it out of pocket, even though a cancer patient is expected to have more than one cycle of treatment with multiple medicine regimens. Calculations were based on an 80 kg adult where needed.

Number of days wage to afford treatment = cost of vial(s) of cancer medicine needed per month /daily wage of lowest paid government worker. The WHO recommends that for a treatment to be affordable it should not exceed 1 day's wages using the wage of the unskilled LPGW [17].

It is important to bear in mind that these costs refer only to the medicine component of the total treatment costs. Consultation fees and diagnostic tests may mean that the total cost to the patient is considerably higher.

No cancer patients were included in this study as it involved data collection. Due to the COVID 19 pandemic, safety aspects that adheres to national directives on social distancing, washing of hands with soap and water, use of hand sanitizers and use of face masks were followed.

Ethical approval for the study was granted by the University of Huddersfield, UK; Ethics Review Committee (ERC) of the Ghana Health Service (GHS), Ghana; and the Scientific and Technical Committees of the Korle Bu Teaching Hospital (KBTH), Ghana; and Komfo Anokye Teaching Hospital (KATH), Ghana respectively. Administrative permission was also obtained from the managers of the medicine's outlets.

Results

Sixty-five (65) individual cancer medicines (LPGs and OBs of different strengths and dosage form) were surveyed in 29 medicine outlets (See Table 2).

Table 2. Cancer Medicines found in facilities, Conditions and Listing on the NEML, WHO EML, WHO EMLc

No.	International Non-Proprietary Name (INN)	Innovator Brand (OB) Name	Branded Generic Name (s)	Medicine Strength	Dosage Form	Listed in Ghana EML [13]	Listed in WHO EML [28]	Listed in WHO EMLc [29]	Chemotherapy Indication (5)
1	Abiraterone	Zytiga	-	250mg	Tab		x		Hormones for prostate cancer.
2	Anastrozole	Arimidex	-	1mg	Tab		x		Hormones and antagonist for breast cancer.
3	Bevacizumab	Avastin,	-	400mg	Vial				Colorectal
4	Bicalutamide	Casodex	-	50mg	Tab	x	x		Hormones and antagonist for prostate cancer.
5	Bicalutamide	Casodex	-	150mg	Tab				Hormones and antagonist for prostate cancer.
6	Bleomycin	Bleo-Kyowa	Bleowel, Bleocel	15 IU PFR	Vial		x	x	Natural product for testis, cervical ovarian, Hodgkin’s disease, non-Hodgkin’s lymphoma.
7	Bortezomib	Velcade	Neomib	3.5mg	Vial		x		Miscellaneous agent for Multiple myeloma and mantle cell lymphoma.
8	Capecitabine	Xeloda	-	500mg	Tab		x		Anti-metabolite for breast, colon, esophageal, stomach, pancreas, premalignant skin lesion (topical), head and neck.

9	Carboplatin	Paraplatin	Carbotin, Carbotinol, Kemocarb	150mg	Vial	x	x	Alkylating agent for testicular, ovarian, bladder, esophageal, lung, colon, breast, brain, neuroblastoma, head, and neck.
10	Carboplatin	Paraplatin	Carbotin, Carbotinol, Kemocarb	450mg	Vial	x	x	Alkylating agent for testicular, ovarian, bladder, esophageal, lung, colon, breast, brain, neuroblastoma, head, and neck.
11	Chlorambucil	Leukeran	Celkeran, Chloramax	2mg	Tab	x		Alkylating agent for chronic lymphocytic leukemia, macroglobulinemia, Hodgkin lymphoma, and non-Hodgkin lymphoma
12	Cisplatin	Platinol	Cistero-10, Abiplatin, Kemoplat	10mg/10 ml	Vial	x	x	Alkylating agent for testicular, ovarian, bladder, esophageal, lung, head and neck, colon, breast, cervical, mesothelioma, brain tumors and neuroblastoma.

13	Cisplatin	Platinol	Cistero-50, Kemoplat, Celplat	50mg	Vial	x	x	x	Alkylating agent for testicular, ovarian, bladder, esophageal, lung, head and neck, colon, breast, cervical, mesothelioma, brain tumors and neuroblastoma.
14	Cyclophosphamide	Endoxan, Cytoxan	Cycloxan, Phoxelon	50mg	Tabs	x	x	x	Alkylating Agent for acute and chronic lymphocytic leukemia, Hodgkin's disease, multiple myeloma, ovarian, breast, small cell lung, neuroblastoma, and sarcoma.
15	Cyclophosphamide	Endoxan, Cytoxan	Phoxelon-500, Cyphos	500mg	Vial	x	x	x	Alkylating Agent for acute and chronic lymphocytic leukemia, Hodgkin's disease, multiple myeloma, ovarian, breast, small cell lung, neuroblastoma, and sarcoma.
16	Cyclophosphamide	Endoxan, Cytoxan	Cyphos	1g	Vial		x	x	Alkylating Agent for acute and chronic lymphocytic leukemia, Hodgkin's disease,

								multiple myeloma, ovarian, breast, small cell lung, neuroblastoma, and sarcoma.
17	Cytarabine	Cytosar-U	Cytalon-100	100mg	Vial	x	x	Anti-metabolite for acute myelogenous and acute lymphocytic leukemia and non-Hodgkin’s lymphoma.
18	Dacarbazine	DTIC-Dome	Celdaz, Dacarex	200mg	Vial			Alkylating agent for malignant melanoma, Hodgkin’s disease, and soft-tissue melanoma.
19	Dacarbazine	DTIC-Dome	Celdaz-500	500mg	Vial			Alkylating agent for malignant melanoma, Hodgkin’s disease, and soft-tissue melanoma.
20	Dactinomycin/ Actinomycin D	Cosmegen	Dacilon	0.5mg	Vial	x	x	Natural product for choriocarcinoma, Wilms’ tumor, rhabdomyosarcoma, testis, Kaposi’s sarcoma, Ewing's sarcoma, trophoblastic neoplasm, and ovarian.
21	Daunorubicin	**C**erubidine	Daunotec	20mg	Vial			Natural product for acute and chronic myelogenous leukemia, and acute

									lymphocytic leukemia, and Kaposi's sarcoma.
22	Docetaxel Trihydrate	Taxotere	Docetero-20	20mg	Vial		x		Natural products for ovarian, breast, lung, prostate, bladder, stomach, head, and neck cancer
23	Docetaxel Trihydrate	Taxotere	Daxotel, Docetero-80, Docetaxel Sandoz	80mg	Vial				Natural products for ovarian, breast, lung, prostate, bladder, stomach, head, and neck cancer
24	Docetaxel Trihydrate	Taxotere	-	120mg	Vial				Natural products for ovarian, breast, lung, prostate, bladder, stomach, head, and neck cancer
25	Doxorubicin HCL	Caelyx, Adriblastina RD, Adriblastina CSV	-	10mg	Vial	x	x	x	Natural product for soft-tissue, osteogenic, and other sarcoma, Breast, bladder, Kaposi's sarcoma, Hodgkin’s disease, non-Hodgkin’s lymphoma, acute lymphocytic leukemia, breast, genitourinary, thyroid, lung, stomach,

									neuroblastoma and other childhood and adult sarcomas.
26	Doxorubicin HCL	Caelyx, Adriblastina RD, Adriblastina CSV	Doxinyl -50, Doxorubicine HCl Sandoz	50mg	Vial		x	x	Natural product for soft-tissue, osteogenic, and other sarcoma, Breast, bladder, Kaposi's sarcoma, Hodgkin's disease, non-Hodgkin's lymphoma, acute lymphocytic leukemia, breast, genitourinary, thyroid, lung, stomach, neuroblastoma and other childhood and adult sarcomas.
27	Epirubicin	Pharmorubicin	Epiget-50, Epiruba	50mg	Vial				Breast
28	Etoposide	Vepesid, Etopophos	Posid, Etopa, Etovel, Oncosid-100	100mg/5 ml	Vial	x	x	x	Natural product for testis, lung, breast cancer, Hodgkin's disease, non-Hodgkin's lymphomas; acute myelogenous leukemia, Kaposi's sarcoma, neuroblastoma, and ovarian.
29	Exemestane	Aromasin	-	25mg	Tab				Hormones and antagonist for breast cancer.

30	Filgrastim	Neupogen, Zarzio, Nivestim, Accofil	-	300mcg	Vial		x	x	Hormonal Immune modulator for prophylaxis in patients at high risk for developing or have developed febrile neutropenia associated with myelotoxic chemotherapy.
31	Fluorouracil	Fluroblastin,	Raciwel	50mg/ml	Vial		x	x	Anti-metabolite for breast, colon, esophageal, stomach, pancreas, cervical, premalignant skin lesion (topical), head and neck.
32	Fluorouracil	Fluroblastin,	Raciwel, Fluracil, 5-flucel	500mg	Vial				Anti-metabolite for breast, colon, esophageal, stomach, pancreas, cervical, premalignant skin lesion (topical), head and neck.
33	Gemcitabine	Gemzar,	Gemget-1000, Gemwel	1000mg	Vial		x		Antimetabolite for pancreatic, ovarian, lung, testicular, breast, and bladder.
34	Goserelin	Zoladex	-	3.6mg	Vial	x			Adjuvant for hormone therapy

35	Goserelin	Zoladex	-	10.8mg	Vial	x			Adjuvant for hormone therapy
36	Hydroxy Urea (hydroxy carbamide)	Hydrea, Siklos	-	250mg	Tabs		x	x	Miscellaneous agent for chronic myelogenous leukemia, polycythemia, cervical, and essential thrombocytosis.
37	Ifosfamide+ Mesna	Haloxan 2G with Uromitexan	-	1g	Vial		x	x	Alkylating Agent for non-Hodgkin's lymphoma, multiple myeloma, neuroblastoma, breast, ovary, lung cancer, Wilms' tumor, cervix, testis, soft-tissue sarcoma, bladder, muscles, and bones.
38	Imatinib	Gleevec, Glivec	Veenat-100	100mg	Tab		x	x	Miscellaneous agent for chronic myelogenous leukemia, gastrointestinal stromal tumors, hyper eosinophilia syndrome.
39	Imatinib	Gleevec, Glivec	-	400mg	Tab		x	x	Miscellaneous agent for chronic myelogenous leukemia, gastrointestinal stromal tumors, hyper eosinophilia syndrome.
40	Irinotecan	Campto	Irinotel	100mg/5 ml	Vial		x	x	Natural product for colon, and small cell lung.

41	L-Asparaginase	Spectrila	Bionase	10,000iu	Vial		x	x	Natural product for acute lymphoblastic leukemia.
42	Lenalidomide	Revlimid	Lenalid-10	10mg	Cap				Miscellaneous agent (immune modulator) for Myelodysplasia (5q− syndrome), multiple myeloma.
43	Leuprolide Acetate	Prostap, Lupron	-	3.75mg	Vial	x			Hormone and antagonist for prostate and breast.
44	Leuprolide Acetate	Prostap, Lupron	Luprova	11.25mg	Vial				Hormone and antagonist for prostate and breast.
45	Liposomal Doxorubicin	Myocet, Doxil, Caelyx	-	20mg	Vial				Natural product for soft-tissue, osteogenic, and other sarcoma, Hodgkin’s disease, non-Hodgkin’s lymphoma, acute leukemia, breast, genitourinary, thyroid, lung, and stomach cancer, neuroblastoma, Kaposi's sarcoma, and other childhood and adult sarcomas.
46	Liposomal Doxorubicin	Myocet, Doxil, Caelyx	-	50mg	Vial		x	x	Natural product for soft-tissue, osteogenic, and other sarcoma, Hodgkin’s disease, non-Hodgkin’s lymphoma, acute

									leukemia, breast, genitourinary, thyroid, lung, and stomach cancer, neuroblastoma, Kaposi's sarcoma, and other childhood and adult sarcomas.
47	Melphalan	Alkeran, Evomela	Alkacel-2	2mg	Tab		x		Alkylating agent for multiple myeloma, ovarian, melanoma, and AL amyloidosis.
48	Mercaptopurine	Puri-Nethol, Xaluprine		50mg	Tab		x	x	Anti-metabolite for acute lymphocytic and chronic myelogenous leukemia, small cell non-Hodgkin’s lymphoma, Crohn's disease, and ulcerative colitis.
49	Mercaptopurine	Puri-Nethol, Xaluprine	-	150mg	Tab				Anti-metabolite for acute lymphocytic and chronic myelogenous leukemia, small cell non-Hodgkin’s lymphoma, Crohn's disease, and ulcerative colitis.
50	Methotrexate	Methofill, Metoject PEN,	Biotrexate	2.5mg	Tab	x	x	x	Antimetabolite for acute lymphocytic leukemia; choriocarcinoma; breast,

		Nordimet, Zlatal						head, neck and lung cancers; osteogenic sarcoma; bladder cancer.
51	Methotrexate	Methofill, Metoject PEN, Nordimet, Zlatal	Methocel-50	50mg	Vial	x	x	Antimetabolite for acute lymphocytic leukemia; choriocarcinoma; breast, head, neck and lung cancers; osteogenic sarcoma; bladder cancer.
52	Mitomycin	Mitocin		10mg	Vial			Natural product for stomach, anal, breast, superficial bladder tumors and lung cancer.
53	Oxaliplatin	Eloxatin		100mg	Vial	x	x	Alkylating agent for testicular, ovarian, bladder, esophageal, lung, colorectal, breast cancer head and neck.
54	Paclitaxel	Taxol, Abraxane	Intaxel, Ataxil, Paclitec-100, Pacliwel, Paclitec-100, Paclitaxel Sandoz	100mg	Vial			Natural products for ovarian, breast, lung, prostate, bladder, esophageal, Kaposi sarcoma, cervical, and pancreatic, head and neck cancer.
55	Sorafenib	Nexavar	Soranib, Orib, Sorafenat	200mg	Tab			Miscellaneous agent for renal, primary kidney cancer, advanced primary

								liver cancer, FLT3-ITD positive AML and radioactive iodine resistant advanced thyroid carcinoma.
56	Tamoxifen	Kessar 10, Nolvadex, Soltamox, Tamoxen	Tamoxifen-Teva	10mg	Tab	x		Hormones and antagonist for breast cancer.
57	Tamoxifen	Nolvadex- D / Kessar 20, Nolvadex, Soltamox, Tamoxen	Cytotam	20mg	Tab	x		Hormones and antagonist for breast cancer.
58	Thalidomide	Talidex	Thalix-50	50mg	Cap	x		Miscellaneous agent (immune modulator) for multiple myeloma.
59	Thalidomide	Talidex	Thalix-100	100mg	Cap			Miscellaneous agent (immune modulator) for multiple myeloma.
60	Trastuzumab	Herceptin,	-	600mg	Vial	x		Breast and stomach
61	Triptorelin Acetate	Decapeptyl	-	3.75mg	Vial			Prostate
62	Vinblastine	Velban	Chemoblast	10mg	Vial	x	x	Natural products for Hodgkin’s disease, non-Hodgkin’s lymphoma, non-small cell lung,

									bladder, brain, melanoma, and testis.
63	Vincristine	Oncovin, Vincasar, Marqibo	Biocristine-AQ, Vincristine Medcrist, Vinlon-1, Vincristine Micristin, Cytocristin	1mg	Vial	x	x	x	Natural products for acute lymphocytic leukemia, acute myeloid leukemia, neuroblastoma, Wilms' tumor, rhabdomyosarcoma; Hodgkin's disease; non-Hodgkin's lymphoma, and small cell lung.
64	Vinorelbine	Navelbine	Vinelbine	50mg	Vial		x	x	Natural products for breast and non-small cell lung.
65	Zoledronic Acid	Zometa	Zoldron, Zelodro-Denk	4mg/5ml	vial		x		Adjuvant for bone diseases.

Availability

Overall availability of the cancer medicines (OB and LPG) in all surveyed facilities was very low, with a median availability of 10.3%, ranging from 3.4% (lowest) to 48.3% (highest). The comparisons between medicine groups and facilities were not statistically significant as $p>0.05$. The LPGs were more available than the OBs in all sectors. The mean availability of the LPGs was highest in the private hospitals (13.08%), then public hospitals (10.55%) with the private pharmacies (9.69%) having the lowest availability (2.42%). In instances, where LPGs were not available, it was highest in private hospitals (49.5%), public hospitals (34.0%) and private

pharmacies (16.5%) respectively (See Table 3, Fig 1a).The OBs were more available than the LPGs in the private hospitals (5.38%). The mean availability of the OBs in the private pharmacies was 2.46% and the public hospitals had the lowest availability (2.42%). In instances, where OBs were not available, it was highest in private hospitals (35.4%), public hospitals (34.1%) and private pharmacies (30.5%) respectively (See Table 3, Figs 1a and 1b).

Table 3. Percentage Availability Data

Facility	OB Mean	LPG Mean
Private Hospital	5.38	13.08
Public Hospital	2.42	10.55
Private Pharmacy	2.46	9.69
All Facilities	3.42	11.11

Figs 1a and 1b. Percentage Availability of LPG and OB using Kruskal Wallis Test.

Out of 65 cancer medicines, 5, 33 and 48 cancer medicines were not available in the private pharmacies, public hospitals, and private hospitals respectively.

In the private pharmacies, the median availability for cancer medicines was 10.0%. For LPGs, it ranged from 5.0% (lowest) to 45.0% (highest) and 5.0% (lowest) to 25.0% (highest) for the OB cancer medicines (See Fig 2a). For the public hospitals, the median availability of cancer medicines was 14.3%. It ranged from 14.3% (lowest) to 85.7% (highest) for LPGs and 14.3% (lowest) to 28.6% (highest) for OB cancer medicines (See Fig 2b). In the private hospitals, the median availability of cancer medicines was 50.0%. It ranged from 50.0% (lowest) to 100.0% (highest) for LPGs and 50.0% (lowest and highest) for OB cancer medicines (See Fig 2c).

16.92% of cancer medicines found in the medicine outlets were listed on the NEML. There were other strengths listed on the NEML, but not available to be surveyed. Absence of a medicine from

the NEML did not necessarily mean it was unavailable, as 83.08% of cancer medicines found in the medicine outlets were not listed on the NEML. The WHO EML and WHO EMLc listed 66.15%

Fig 2a. Availability of Cancer Medicines (OB and LPG) in Private Pharmacies

Fig 2b. Availability of Cancer Medicines (OB and LPG) in Public Hospitals

Fig 2c. Availability of Cancer Medicines (OB and LPG) in Private Hospitals

Price Ratio Comparisons

The 65 sampled cancer medicines (OBs and LPGs) showed significant price differences between their HPM and LPM (See Table 4). The price comparisons were not statistically significant as $p>0.05$.

The mean of median prices (USD) of the LPGs was highest in the private hospitals (55.42), followed by the public hospitals (32.10) with the lowest being the private pharmacies (31.99). The prices ranged from a minimum of 0.25 to a maximum of 227.98 The highest median price was found in private pharmacies (227.98), private hospitals (165.20) and the lowest in public hospitals (132.16) (See Table 4, Figs 3a and 3b).

The mean of median prices (USD) of the OBs was highest in the private hospitals (391.39), then public hospitals (120.19) with the lowest being the private pharmacies (104.67). The median prices ranged from a minimum of 0.41 to a maximum of 1321.60. The highest median price was found in private hospitals (1321.60), public hospitals (646.59) and the lowest in private pharmacies (581.21) (See Table 4, Figs 3a and 3b).

Table 4. Median Price (USD) Variations between HPM, LPM, OB, LPG

Facility Price	Mean of Median LPG	Minimum of Median LPG	Maximum of Median LPG	Mean of Median OB	Minimum of Median OB	Maximum of Median OB
Private Hospital	55.42	2.97	165.20	391.39	0.41	1321.60
Public Hospital	32.10	0.48	132.16	120.19	1.43	646.59
Private Pharmacy	31.99	0.25	227.98	104.67	0.50	581.21
All facilities	35.59	0.25	227.98	169.72	0.41	1321.60

Figs 3a and 3b. Median Price Variations between OB and LPG using Kruskal Wallis Test

In the public hospitals, 8.33% of the cancer medicines price differential was above 50%, with a cost differential ratio higher than 2. Bicalutamide 150 mg tablets (95.15%) and Vincristine 1mg vial (5.56%) had the highest and lowest price differentials respectively (See Table 5). In the private pharmacies, 77.78% had a price ratio <3 and 33.33% of the cancer medicines had a price variation ≥50%. Thalidomide 50 mg capsules (97.92%) had the highest price differential and Leuprolide acetate 11.25mg vial had the lowest price differential (4.27%) (See Table 6). The private hospitals showed that 66.67% of their medicines had cost differentials above 50%, and ratios higher than 2. Doxorubicin HCL 50mg vial (88.75%) and Docetaxel Trihydrate 80mg vial (21.67%) had the highest and lowest cost differential respectively (See Table 7). Overall, none of the medicines had a price ratio<1, suggesting inefficient procurement of cancer medicines in Ghana.

Fig 4 shows the median price variability/cost differences between 6 OBs and 6 LPG from public hospitals and private pharmacies. Only those medicines for which both the originator brand and a

generically equivalent product were found, were included in the analysis to allow for the comparison of prices between the two product types.

The lowest OB/LPG price variation was chlorambucil 2mg tablet (21.05%) and the highest was Epirubicin 50mg vial (44.23%) respectively. Cyclophosphamide 50mg tablets showed the highest negative price variation of-566.67%. Liposomal Doxorubicin 50mg vial, Liposomal Doxorubicin 20mg vial, Epirubicin 50mg vial showed negative price variations of -325.00%, -166.67% and -9.09% respectively, as these LPGs, were more expensive when compared to the OBs, suggesting efficient pricing of the OBs. Some OBs were more expensive than the LPGs, with MPRs of 1.27 and 1.97 respectively.

Table 5. Comparison of the Lowest Price with the Highest Price of the same Medicine in Public Hospitals:

**No.**	**Medicine Name**	**Medicine Strength**	**Dosage Form**	**Target Pack Size**	**Medicine Type**	**Minimum (USD)**	**Maximum (USD)**	**Cost differential between Min and Max (%)**	**Price Ratio**
1	Bicalutamide (Casodex)	150mg	tabs	1	OB	4.81	99.12	95.15	20.60
2	Carboplatin (Carbotin, Carbotinol, Kemocarb)	150mg	vial	1	LPG	21.48	32.05	32.99	1.49
3	Carboplatin (Carbotin, Carbotinol, Kemocarb)	450mg	vial	1	LPG	58.15	79.63	26.97	1.37
4	Cisplatin (Cistero-50, Kemoplat, Celplat)	50mg	vial	1	LPG	8.26	15.53	46.81	1.88
5	Cyclophosphamide (Phoxelon-500, Cyphos)	500mg	vial	1	LPG	2.64	3.30	20.00	1.25

6	Doxorubicin HCL (Doxinyl -50, Doxorubicine HCl Sandoz)	50mg	vial	1	LPG	14.04	16.52	15.00	1.18
7	Etoposide (Posid, Etopa, Etovel, Oncosid-100)	100mg	vial	1	LPG	5.62	6.94	19.05	1.24
8	Fluorouracil (Raciwel, Fluracil, 5-flucel)	500mg	vial	1	LPG	1.98	2.31	14.29	1.17
9	Goserelin (Zoladex)	10.8mg	vial	1	OB	223.02	289.35	22.92	1.30
10	Methotrexate (Methocel-50)	50mg	vial	1	LPG	5.45	5.95	8.33	1.09
11	Paclitaxel (Intaxel, Ataxil, Paclitec-100, Pacliwel, Paclitec-100, Paclitaxel Sandoz)	100mg	vial	1	LPG	32.21	37.17	13.33	1.15
12	Vincristine (Biocristine-AQ, Vincristine Medcrist, Vinlon-1, Vincristine Micristin, Cytocristin)	1mg	vial	1	LPG	2.81	2.97	5.56	1.06

Table 6. Comparison of the Lowest Price with the Highest Price of the same Medicine in Private Pharmacies

**No.**	**Medicine Name**	**Medicine Strength**	**Dosage Form**	**Target Pack Size**	**Medicine Type**	**Minimum (USD)**	**Maximum (USD)**	**Cost differential between Min and Max (%)**	**Price Ratio**
1	Abiraterone (Zytiga)	250mg	tabs	1	OB	4.79	6.20	22.67	1.29
2	Anastrozole (Arimidex)	1mg	tabs	1	OB	1.07	1.72	37.46	1.60

3	Bleomycin (Bleowel, Bleocel)	15 IU PFR	vial	1	LPG	23.95	28.91	17.14	1.21
4	Capecitabine (Xeloda)	500mg	tabs	1	OB	2.15	2.81	23.53	1.31
5	Carboplatin (Carbotin, Carbotinol, Kemocarb)	150mg	vial	1	LPG	16.52	82.68	80.02	5.01
6	Carboplatin (Carbotin, Carbotinol, Kemocarb)	450mg	vial	1	LPG	24.12	79.63	69.71	3.30
7	Chlorambucil (Celkeran, Chloramax)	2mg	tabs	1	LPG	1.98	7.10	72.09	3.58
8	Cisplatin (Cistero-10, Abiplatin, Kemoplat)	10mg	vial	1	LPG	14.87	19.33	23.08	1.30
9	Cisplatin (Cistero-50, Kemoplat, Celplat)	50mg	vial	1	LPG	13.22	18.17	27.27	1.38
10	Cyclophosphamide (Cyphos)	1g	vial	1	LPG	3.63	18.50	80.36	5.09
11	Cyclophosphamide (Cycloxan, Phoxelon)	50mg	tabs	1	LPG	0.26	3.47	92.38	13.13
12	Cyclophosphamide (Phoxelon-500, Cyphos)	500mg	vial	1	LPG	2.97	4.96	40.00	1.67
13	Cytarabin (Cytalon-100)	100mg	vial	1	LPG	9.91	17.35	42.86	1.75
14	Docetaxel Trihydrate (Docetero-20)	20mg	vial	1	LPG	42.29	46.26	8.57	1.09
15	Docetaxel Trihydrate (Daxotel, Docetero-80, Docetaxel Sandoz)	80mg	vial	1	LPG	92.51	111.51	17.04	1.21

16	Doxorubicin HCL (Doxinyl - 50, Doxorubicine HCl Sandoz)	50mg	vial	1	LPG	13.22	18.17	27.27	1.38
17	Etoposide (Posid, Etopa, Etovel, Oncosid-100)	100mg	vial	1	LPG	6.44	9.09	29.09	1.41
18	Exemestane (Aromasin)	25mg	tabs	1	OB	1.38	9.17	84.97	6.65
19	Fluorouracil (Raciwel, Fluracil, 5-flucel)	500mg	vial	1	LPG	1.90	3.30	42.50	1.74
20	Gemcitabine (Gemget-1000, Gemwel)	1000mg	vial	1	LPG	94.99	132.16	28.13	1.39
21	Goserelin (Zoladex)	3.6mg	vial	1	OB	109.28	194.11	43.70	1.78
22	Goserelin (Zoladex)	10.8mg	vial	1	OB	264.32	341.65	22.63	1.29
23	Hydroxyurea (Hydrea, Siklos)	250mg	tabs	1	OB	0.41	0.83	50.00	2.00
24	Leuprolide Acetate (Luprova)	11.25mg	vial	1	LPG	173.96	181.72	4.27	1.04
25	Melphalan (Alkacel-2)	2mg	tabs	1	LPG	2.51	3.30	24.00	1.32
26	Methotrexate (Biotrexate)	2.5mg	tabs	1	LPG	0.12	0.38	69.57	3.29
27	Methotrexate (Biotrexate Methocel-50)	50mg	vial	1	LPG	6.61	8.26	20.00	1.25
28	Mitomycin	10mg	vial	1	LPG	32.21	43.12	25.29	1.34
29	Paclitaxel (Intaxel, Ataxil, Paclitec-100, Pacliwel, Paclitec-100, Paclitaxel Sandoz)	100mg	vial	1	LPG	23.95	39.65	39.58	1.66
30	Sorafenib (Soranib, Orib, Sorafenat)	200mg	tabs	1	LPG	1.82	4.30	57.69	2.36
31	Tamoxifen (Cytotam)	20mg	tabs	1	LPG	0.33	0.79	58.33	2.40

32	Thalidomide (Thalix-50)	50mg	caps	1	LPG	1.16	55.51	97.92	48.00
33	Thalidomide (Thalix-100)	100mg	cap	1	LPG	1.82	2.48	26.67	1.36
34	Trastuzumab	600mg	vial	1	OB	563.75	598.68	5.84	1.06
35	Vincristine (Biocristine-AQ, Vincristine Medcrist, Vinlon-1, Vincristine Micristin, Cytocristin)	1mg	vial	1	LPG	2.97	6.61	55.00	2.22
36	Zoledronic Acid (Zoldron, Zelodro-Denk)	4mg/5ml	vial	1	LPG	39.32	54.52	27.88	1.39

Table 7. Comparison of the Lowest Price with the Highest Price of the same Medicine in Private Hospitals

No.	Medicine Name	Medicine Strength	Dosage Form	Target Pack Size	Medicine Type	Minimum (USD)	Maximum (USD)	Cost differential between Min and Max (%)	Price Ratio
1	Cyclophosphamide (Cyphos)	1g	vial	1	LPG	4.13	11.56	64.29	2.80
2	Docetaxel Trihydrate (Daxotel, Docetero-80, Docetaxel Sandoz)	80mg	vial	1	LPG	116.47	148.68	21.67	1.28
3	Doxorubicin HCL (Doxinyl - 50, Doxorubicine HCl Sandoz)	50mg	vial	1	LPG	3.72	33.04	88.75	8.89

Fig 4. Comparison of OB with LPG of the same Medicine- Public Hospitals and Private Pharmacies

The MPR analysis included medicines with prices found for both types OB and LPG. The inflation adjusted MPRs for LPGs and OBs across Public Hospitals and Private Pharmacies ranged from

0.01 to 10.5 and from 0.03 to 1.66, respectively (See Figs 5 and 6). Only 10.34% and 6.52% of medicines in Public Hospitals and Private Pharmacies respectively had an MPR more than.

Fig 5. MPR of Medicine (OB and LPG)-Public Hospitals

Fig 6. MPR of Medicine (OB and LPG)-Private Pharmacies

Affordability

Affordability was assessed for all the cancer medicines (OB and LPG) using the daily wage of the unskilled LPGW and found to cost more than 1 day's wage. There were differences in affordability in all the sectors, but they were not statistically significant as p>0.05.

When comparing sectors, the affordability of all the cancer medicines is better in public hospitals (54.81). The private hospitals (105.64) are less expensive than the private pharmacies (134.83). The affordability of OBs and LPGs are more in the public hospitals (188.00 and 54.81 respectively) followed by the private hospitals (222.06 and 105.64 respectively), with the private pharmacies being the most expensive (653.57 and 134.83) respectively (See Table 8 and Figs 7a and 7b).

Table 8. Affordability of OB and LPG in all Sectors

**Affordability**	**Mean LPG**	**Mean OB**
Private Hospital	105.64	653.57
Public Hospital	54.81	188.00
Private Pharmacy	134.83	222.06
All Facilities	103.42	304.30

Figs 7a and 7b: Affordability of OB and LPG using Kruskal Wallis Test.

In all the sectors, one-month treatment with medicines which were most expensive requiring several days wages were, Thalidomide 50mg capsules (LPG), 1642 days' wages and is the most unaffordable. Bevacizumab 150mg vial (OB) and Bicalutamide 150mg tablets (OB) required 1590- and 753-days wages respectively. Methotrexate 2.5mg tablets (LPG), Liposomal Doxorubicin 50mg vial (OB), and Bicalutamide 50mg tablets (LPG) required 1.4, 1.6- and 6.9-days wages respectively (See Fig 8, 9, 10).

All the OB treatments, costs more than 1 day's wage, with Bevacizumab 400mg vial being the most expensive requiring 1590 days wages followed by Bicalutamide 150 mg vial which required 753 days wages and Trastuzumab 600mg vial required 602 days wages, whilst the most affordable was Liposomal Doxorubicin 50mg vial which required 1.6 days wages (See Fig 8, 9,10).

For the LPGs treatment costs, they all required more than a day's wage, with Thalidomide 50mg capsules being the most unaffordable requiring 1642 days wages, followed by Imatinib 400mg tablets requiring 575 days wages and Bortezomib 3.5mg vial requiring 330 days wages. The most affordable LPG was Methotrexate 2.5mg tablets which required 1.4 days wages (See Fig 8, 9,10).

For the OBs with a comparator LPG, the cost of a one-month treatment with Bicalutamide 150mg tablets (OB) required about 753 days' wages and 8-days' wages for the LPG.

There were some instances where the OBs were more affordable than their comparator LPGs, e.g. for Cyclophosphamide 50mg tablets, people have to work for 289 days to pay for the LPG, whereas they had to work for 43 days to pay for the OB. It is important to bear in mind that these costs refer only to the medicine component of the total treatment costs. Consultation fees and diagnostic tests may mean that the total cost to the patient is considerably higher.

Fig 8. Affordability of Cancer Medicines in Public Hospitals based on day's wages

Fig 9. Affordability of Cancer Medicines in Private Pharmacies based on day's wages

Fig 10. Affordability of Cancer Medicines in Private Hospitals based on day's wages

Discussion:

The results of this study show that cancer medicine prices in Ghana are high, and there are large price differences amongst the sectors, between HPM and LPM, as well as between OB and LPG. The private sectors (private pharmacy and private hospitals) had the most expensive LPG and OB prices compared to the public sector (public hospitals). The private pharmacies had the lowest OB median prices, because of some pharmacies being suppliers/wholesalers of cancer medicines to both public and private pharmacies. Whilst the public hospitals had the lowest LPG median prices probably due to government policies on generic prescribing and medical insurance reimbursements. The price variability between the OB and LPG, showed that when some OB medicines are prescribed/dispensed in Ghana, patients pay more than they would pay for generics. However, some LPGs were more expensive than the OBs showing efficient generic procurement and pricing probably due to generic competition, price-fixing, market competition or some other factors [28].

Similar findings were also seen in studies conducted in LMICs (Countries; Nepal, India, Malaysia, and Regions; Africa, Latin America, South East Asia, Western Pacific, East Mediterranean) on pricing of cancer medicines [7, 31, 28-30, 32-35]. These published studies on the pricing of both adult and pediatric cancer medicines showed wide variations in prices in the individual and medicine categories and between brands. The price variation in public versus private facilities was also evident [30, 32, 33, 35-39].

High patient prices can be due to OB patent protection, lack of generic competition, suppliers of generic medicines pricing popular products only slightly below the originator brand version, high manufacturer profit margins, high government taxes and duties on medicines, inefficient supply system and high wholesale or retail mark-ups [39]. Differences pricing policies account for the

varying prices of medicines [40]. The existence of generics on the market might affect originator prices in some countries, whereas in other countries originator prices remained at a high level [41]. Cancer care in Ghana is hampered by several factors including high cost of medicines and little reimbursement by insurance. In Ghana, only cervical and breast cancer medicines are eligible for reimbursement under the NHIS [42]. It is based on the median generic price, thus forcing dispensers to seek and dispense only generics. Patients pay for most cancer medicines out-of-pocket and face financial hardship due to unaffordable medical cost. This may lead to premature termination or interruption in treatment [43]. Many medicine purchases by the hospitals are small in volume and, therefore, high in price.

Over 85% of OB/LPG medicines in the Public Hospitals (89.66%), Private Pharmacies (91.30%) and Private Hospitals (88.24%) were moderately inexpensive relative to the IRP, and may suggest effective procurement, or compromised quality control etc. [23]. but this cannot be ascertained as there are some ambiguities when making international comparisons, due to varying medicine components, such as market size, market penetration pricing mechanisms, scale of economy, and taxation [25]. Further research to investigate reasons behind this variation is needed.

Ghana has a NMP to improve medicine pricing to ensure affordability [7]. This may not be strictly implemented as the government sets the price for the NHIS cancer medicines only. Importation of all cancer medicines, is largely affected by forex fluctuations and determined by the pharmaceutical wholesalers/suppliers based on their production, marketing cost, taxes, duties and desired benefit (30-40% for taxes and tariffs, and 50-200% for markups) [7].

Government of Ghana (GoG) should launch initiatives to empower patients to shop around for cheaper medicine prices, promote quality assured generics and generic prescribing by physicians,

improve pricing and price transparency, support cost sharing, encourage value based reimbursement by medical insurance companies, support local manufacturing and companies that manufacture or supply cheap cancer medicines with tax incentives and grant faster approvals to new cancer medicines [10, 32, 41, 44, 45]. Price policies and regulations such as tiered pricing, value-based pricing, cost plus pricing, External Reference Pricing (ERP) or International Reference Pricing (IRP) and mark-up regulation across the supply and distribution chain should be implemented to restrain medicine costs [33, 36, 45].

Strong country pharmaceutical pricing policies can improve the affordability of pharmaceutical products when carefully planned, carried out, regularly checked, and revised according to changing conditions [45].

Affordability of cancer medicines in Ghana is a big challenge as all treatments using standard regimens costs more than 1 day’s wage and could pose problems for public health. Cancer medicines are more affordable in the public sector (public hospitals) than in the private sectors (private pharmacies and private hospitals). The LPGW need 1590- and 1642-days wages to afford a month’s treatment with Bevacizumab 400mg vial (OB) and Thalidomide 50mg capsules (LPG), costing 1558.98 USD and 3399.82 USD respectively. This shows that a month’s wage would be woefully inadequate to afford cancer medicines. The results of our findings are consistent with studies showing patterns of affordability amongst countries, unaffordability of LPGs and OBs (adult and pediatric) in public and private facilities, and in some instances leading to treatment abandonment [27, 37, 40, 46, 47].

Most patients in Ghana cannot afford the cancer medicines because of poverty, high inflation, and increasing cost of living. They sometimes present late for treatment, succumb to using herbal preparations and seek traditional spiritual remedies. In developing countries like Ghana, where the

medical insurance is in a developing stage, higher direct out of pocket payments costs means a decreased adherence to therapy, or default on treatment with its resulting disastrous implications and devastating impoverishing effects for families and households (48). The GoG should consider implementing policies that modify current high prices, fund cancer (adult and pediatric) medicines within the national budget, streamlines public and private sector procurement and supply systems to ensure equitable affordability in Ghana [39,46].

In general, the availability of essential medicines has been problematic in sub-Saharan Africa, more than in any other region [49]. The average availability of essential medicines in sub-Saharan Africa is around 40% in the public sector and 60% in the private sector, substantially below the WHO target of 80% medicines availability in all sectors [50].

Our study showed very low availability of the cancer medicines in Ghana. It was disconcerting to see that none of the surveyed cancer medicines met the ideal availability WHO benchmark of 80% in all sectors [51]. The LPGs were more available than the OBs in all sectors and was more available in hospitals than in pharmacies. Conversely, the OBs were more available in the private facilities than the public facilities. It can be reasonably interpreted that patients had to purchase expensive OBs in most cases, compromising the affordability of patients. In Ghana, there are limited suppliers of cancer medicines due to the huge cost associated with the acquisition and supply. Most pharmacies do not routinely stock cancer medicines unless specifically requested for by prescribers, thus having unreliable availability even for those who can afford. There have been cases of stock outs of cancer medicines leading to frustration amongst patients coupled with loss of confidence in the health care system. Other reasons for the poor availability of cancer medicines such as budgetary constraints, poor estimation of demands, delays in receiving the medication orders, in-country distribution challenges, lack of supplier motivation, and in accurate

determination of needs should be addressed [52, 53]. Low availability of cancer medicines in public hospitals forces the patients to seek the private sector and this highlights the need to streamline cancer medicine procurement, distribution, and supply systems in the public system [37, 54].

Our findings are consistent with studies in LMICs showing disparities in the actual availability and formulary availability of essential cancer medicines [27, 41, 52, 55, 56]. The WHO's EML for adults and EMLc for children is a guide to help countries develop their NEML and reimbursable list for the public sector, to improve the availability of adult and childhood cancers medicines respectively [7].While not a direct measure of availability, listing is an important step, to guide procurement and ensure availability of the cancer medicines [56]. An up-to-date list of the Ghana EML and health insurance lists should be maintained. At least for cytotoxic agents, there seems little value in maintaining separate NEMLs for adults and children as the same medicines are used in each group [55, 56].

Africa including Ghana's health challenges are numerous and wide-ranging [57]. With many factors affecting access to cancer medicines [22, 58, 52]. A challenge in cancer medicines is to ensure global and local access to cancer medicines, while maintaining incentives for future research to improve the health outcomes for cancer patients around the world [47].

Conclusion:

Cancer medicine prices in Ghana are high, with large price differences. Availability was particularly very low for the cancer medicines and most patients cannot afford the cancer medicines. There should be a balance in having prices that are affordable, will not impede access, and be available to ensure continuity of supply through healthy pharmaceutical markets. Ghana must address determinants of health holistically with comprehensive policies and multifaceted

interventions and regulations that modify current high prices, provides tax incentives, health insurance and use of generics. This is to ensure equitable availability, timely affordable access, and appropriate use of quality cancer medicines irrespective of ability to pay at the point of use, to achieve healthy lives in line with the Ghana National Health Policy 2020.

Limitations

This study, using basic indicators only, cannot give a complete picture of the pharmaceutical sector in Ghana. International price comparisons include many assumptions that affects its outcome, and thus are only pointers for further investigation. The MPRs were calculated using the only available and outdated 2015 MSH IRPs, with prices deflated using the CPI to get a reliable MPR calculation. To resolve this issue, the HAI experts suggest avoiding calculating it and rely on MUPs only, thus we have presented the MUPs also. Results on affordability may also lead to over-estimation since the calculation used was based on the LPGWs wages. A significant proportion of the population especially patients with jobs in the informal sector earn less than the LPGW. The exclusion of the accompanying cost factors that play a role in the final cost to the cancer patient such as dispensing fees, facility fees, administration fees, doctors' fees means that patients pay more. Use of retail prices, which include add-ons such as taxes and distribution fees was not estimated to identify potential targets for price reduction. Availability had a limitation of not capturing the pattern of medicine availability over time as it was measured at 'one time' on the day of data collection from the health facilities. Availability may be better understood through a longitudinal study instead of a cross-sectional study.

Abbreviations

Ghana (Gh), World Health Organization (WHO), Health Action International (HAI), U.S dollars (USD), Originator Brand (OB), Lowest Priced Generic (LPG), Median Unit Price (MUP), International Reference Price (IRP), Median Price Ratio (MPR), Lowest-Paid Government Worker (LPGW), National Health Insurance Scheme (NHIS), low- and middle-income countries (LMICs), Essential Medicines List (EML), National Essential Medicines List (NEML), National Medicines Policy (NMP), Standard Treatment Guidelines (STG), Essential Medicines list for Children (EMLc), International Nonproprietary Name (INN), Electronic Medicines Compendium (emc), National Comprehensive Cancer Network (NCCN), Management Sciences for Health (MSH), Consumer Price Indices (CPI), International Price Ratio (IPR), Highest-Priced Medicine (HPM) and Lowest-Priced Medicine (LPM), Ethics Review Committee (ERC), Ghana Health Service (GHS), Korle Bu Teaching Hospital (KBTH), Komfo Anokye Teaching Hospital (KATH), Government of Ghana (GoG), External Reference Pricing (ERP), International Reference Pricing (IRP).

Acknowledgements

We thank Divine Darlington Logo (HRU, GHS), for his support in data collection. We thank all personnel, institutions, pharmacies, and hospitals that provided access to their facilities during data collection. We thank Dr. Margaret Ewen (HAI/WHO), Dr Syed Hasan (University of Huddersfield), and Dr Amna Saeed (Lahore Medical and Dental College) for their support.

References

1. Ferlay J, Ervik M, Lam F, Colombet M, Mery L. et al. Global Cancer Observatory: Cancer Today. Lyon: International Agency for Research on Cancer; 2020. Available from: https://gco.iarc.fr/today, Accessed 09 August 2021.
2. World Health Organization. Facts Sheet. 2021. Available from: http://www.who.int/mediacentre/factsheets/fs297/en/ Accessed 09 August 2021.
3. Babar, ZUD. Ten recommendations to improve pharmacy practice in low and middle-income countries (LMICs). *J of Pharm Policy and Pract* 14, 6 (2021). https://doi.org/10.1186/s40545-020-00288-2.
4. Dent J, Manner C.K, Milner D, Mutebi M, Ng'ang'a A, et al. Africa's Emerging Cancer Crisis: A Call to Action. 2017.
5. Howard DH, Chernew ME, Abdelgawad T, Smith GL, Sollano J, et al. New anticancer drugs associated with large increases in costs and life expectancy. Health Aff (Millwood). 2016 Sep 1;35(9):1581-7.
6. Ghana National Medicines Policy 3rd Edition, Ministry of Health. editor: Ghana National Drugs Programme, Ghana. 2017.
7. Minimum Wage. Available from: Ghana's National Daily Minimum Wage for 2021 is GH¢12.53 | News Ghana. Accessed 02 August 2021.
8. Cherny NI, Sullivan R, Torode J, Saar M, Eniu A. ESMO International Consortium Study on the availability, out-of-pocket costs, and accessibility of antineoplastic medicines in countries outside of Europe. Ann Oncol. 28(11):2633–47. 2017.
9. Ministry of Health Facilities, Ghana. Available from: Agencies - Ministry Of Health (moh.gov.gh). Accessed 02 August 2021.

10. Boyle P, Ngoma T, Sullivan R, Ndlovu N, Autier P, et al. Medical Oncology in Africa. The State of Oncology in Africa 2015. iPRI Scientific Publication 4, iPRI, Lyon, France. 2016.

11. Ghana National Drugs Programme. Essential Medicines List, 7th Edition, 2017. Ministry of Health. Ghana. 2017.

12. Babar ZUD. Equitable Access to High-Cost Pharmaceuticals (1st Edition). 2018. Available from: https://www.elsevier.com/books/equitable-access-to-high-cost-pharmaceuticals/babar/978-0-12-811945-7. Accessed 08 June 2021.

13. Essential Medicines List, 7th Edition, 2017. Ministry of Health. editor: Ghana National Drugs Programme, Ghana. 2017.

14. Ghana National Drugs Programme. Standard Treatment Guidelines, Seventh Edition (7th), 2017. Ministry of Health. Ghana. 2017.

15. Renner L, Shah S, Bhakta N, Denburg A, Horton S, et al. Evidence from Ghana Indicates That Childhood Cancer medicines in Sub-Saharan Africa Is Very Cost Effective: A Report from the Childhood Cancer 2030 Network. 2018. Journal of Global Oncology.

16. Ocran Mattila P, Ahmad R, Hasan SS and Babar Z-U-D (2021) Availability, Affordability, Access, and Pricing of Anti-cancer Medicines in Low- and Middle-Income Countries: A Systematic Review of Literature. Front. Public Health 9:628744. doi: 10.3389/fpubh.2021.628744.

17. World Health Organization, Health Action International. Measuring medicine prices, availability, affordability, and price components. 2nd edition. Geneva: World Health Organization; 2008. Updated July 2020. Available from: https://haiweb.org/what-we-

do/price-availability-affordability/collecting-evidence-on-medicine-prices-availability/. Accessed:13 August 2021.

18. Ghana Statistical Service. Available from: https://www.statsghana.gov.gh/regionalpopulation.php?population=MTI5MzE3OTU5OC40NDg1&&Ashanti&regid=1. Accessed 14 July 2021

19. Brunton L, Chabner B, knollman B. Goodman & Gilman's. The Pharmacological Basis of Therapeutics. 12th Edition, 2011.

20. Currency Conversion. Available from: Convert Ghanaian Cedis (GHS) to US Dollars (USD) - Exchange Rates (exchange-rates.org). Accessed 06 October 2021.

21. The electronic medicines compendium (emc). Available from: https://www.medicines.org.uk/emc . Accessed 10 August 2021.

22. National Comprehensive Cancer Network clinical guidelines in practical oncology (NCCN Guidelines), 2020. Available from : https://www.nccn.org/professionals/physician_gls/recently_updated.aspx. Assessed 10 August 2021.

23. International Medical Products Price Guide (2015). Management Sciences for Health. Available from: International Medical Products Price Guide | Management Sciences for Health (msh.org). Accessed 07 October 2021.

24. Consumer Price Index. Available at Ghana Statistical Services(statsghana.gov.gh). Accessed 10 October 2021

25. Saeed, A., Saeed, H., Saleem, Z., Fang, Y., and Babar, Z.-U.-D. (2019). Evaluation of prices, availability, and affordability of essential medicines in Lahore Division, Pakistan:

a cross-sectional survey using WHO/HAI methodology. PLoS One 14, e0216122. doi: 10.1371/journal.pone.0216122.

26. Song, Y., Bian, Y., and Zhen, T. (2018). Making medicines more accessible in China: an empirical study investigating the early progress of essential medicine system. PLoS One 13. doi: 10.1371/journal.pone.0201582.
27. Babar, Z. U., Ibrahim, M. I., Singh, H., Bukahri, N. I., and Creese, A. (2007). Evaluating drug prices, availability, affordability, and price components: implications for access to drugs in Malaysia. PLoS Med. 4, e82. doi: 10.1371/journal.pmed.0040082
28. World Health Organization Model List of Essential Medicines, 22nd List, 2021. Geneva: World Health Organization; 2021.
29. World Health Organization Model List of Essential Medicines for Children, 8th List, 2021. Geneva: World Health Organization; 2021.
30. Shrestha S, Poudel RS, Bhuvan KC, Poudel BK, Sapkota B, Sharm S, Khadka A. Price variation among different brands of cancer medicines available in hospital pharmacies of Nepal. Journal of Pharmaceutical Policy and Practice 2020; 13:6.
31. Management Sciences for Health (MSH), International Medical Products Price Indicator Guide. Cambridge, MA: MSH; 2016. Available from: https://mshpriceguide.org/en/home/. Accessed 19 August 2020.
32. Kolasani B. P., Malathi D. C, Ponnaluri R.R. Variation of Cost among Cancer Medicines Available in Indian Market. 2016. DOI: 10.7860/JCDR/2016/22384.8918.
33. Cuomo R.E., Seidman R.L, Mackey T.K. Country and regional variations in purchase prices for essential cancer medication. 2017. DOI 10.1186/s12885-017-3553-5.

34. Mattila, P.O., Babar, ZUD. & Suleman, F. Assessing the prices and affordability of oncology medicines for three common cancers within the private sector of South Africa. BMC Health Serv Res 21, 661 (2021). https://doi.org/10.1186/s12913-021-06627-6.
35. Babar, Z. U., Ibrahim, M. I., Singh, H., Bukahri, N. I., and Creese, A. (2007). Evaluating drug prices, availability, affordability, and price components: implications for access to drugs in Malaysia. PLoS Med. 4, e82. doi: 10.1371/journal.pmed.0040082
36. Salmasi S, Lee KS, Ming LC, Neoh CF, Elrggal ME, Babar ZU, Khan TM, Hadi MA. Pricing appraisal of cancer medicines in the South East Asian, Western Pacific and East Mediterranean Region. BMC Cancer, 2017; 17: 903.
37. Faruqui N., Martiniuk A., Sharma A., Sharma C., Rathore B., Arora R.S, Joshi R. Evaluating access to essential medicines for treating childhood cancers: a medicines availability, price and affordability study in New Delhi, India. 2018. BMJ Glob Health 2019;4: e001379. doi:10.1136/bmjgh-2018-001379.
38. Gelband H, Sankaranarayanan R, Gauvreau CL, Horton S, Anderson BO, et al. Costs, affordability, and feasibility of an essential package of cancer control interventions in low-income and middle-income countries: key messages from Disease Control Priorities, 3rd edition. Lancet 2016; 387(10033):2133-2144.
39. Mattila, P.O., Babar, ZUD. & Suleman, F. Assessing the prices and affordability of oncology medicines for three common cancers within the private sector of South Africa. BMC Health Serv Res 21, 661 (2021). https://doi.org/10.1186/s12913-021-06627-6.
40. A. Islam, A. Akhter, T. Eden. Cost of treatment for children with acute lymphoblastic leukemia in Bangladesh. 2015. Journal of Cancer Policy 6 (2015) 37–43.

41. Cherny NI, Sullivan R, Torode J., Saar M., Eniu A. The European Society for Medical Oncology (ESMO) European Consortium Study on the availability, out-of-pocket costs and accessibility of antineoplastic medicines in Europe. Annals of Oncology 27: 1423–1443, doi:10.1093/annonc/mdw213. 2016.

42. Ghana National Health Insurance Scheme. Available from: http://www.nhis.gov.gh/ Accessed 25 September 2021.

43. Khatiwoda SR, Dhungana RR, Sapkota VP, Singh S. Estimating the direct cost of Cancer in Nepal: a cross-sectional study in a tertiary Cancer hospital. Front Public Health. 2019; 7:160.

44. S. Mayor, WHO includes 16 new cancer drugs on list of essential medicines. The Lancet Oncology 2015 Jul;16(7):757. doi: 10.1016/S1470-2045(15)70240-8. Epub 2015 May 21.

45. World Health Organization. WHO guideline on country pharmaceutical pricing policies, second edition. 2020. Available from: https://apps.who.int/iris/handle/10665/335692. Accessed 24 September 2021.

46. Sarwar MR, Iftikhar S, Saqib A. Availability of cancer medicines in public and private sectors, and their affordability by low, middle, and high-income class patients in Pakistan. BMC Cancer 2018;18[1]:14.

47. Goldstein DA, Clark J, Tu Y, Zhang J, Fang F, et al. A global comparison of the cost of patented cancer medicines in relation to global differences in wealth. Oncotarget. 2017 May 9;8[42]:71548-71555.

48. Niëns L.M., Brouwer W.B.F Measuring the affordability of medicines: Importance and challenges. Health Policy 112 (2013) 45– 52.

49. Cameron A, Ewen M, Ross-Degnan D, Ball D, Laing R. Medicine prices, availability, and affordability in 36 developing and middle-income countries: a secondary analysis. Lancet 2009; 373:240e9.

50. World Health Organization. Medium-term strategic plan 2008-2013. Geneva: World Health Organization; 2009. Available from: http://apps.who.int/gb/ebwha/pdf_files/ MTSP2009/MTSP1-en.pdf. Accessed 24 September 2021.

51. World Health Assembly. Follow-up to the Political Declaration of the High-level Meeting of the General Assembly on the Prevention and Control of Non-communicable Diseases. Sixty-Sixth World Health Assembly. 2013. Available from: http://apps.who.int/gb/ebwha/pdf_files/WHA66/A66_R10-en.pdf?ua=1. Accessed 28 September 2021.

52. Cherny NI, Sullivan R, Torode J., Saar M., Eniu A. The European Society for Medical Oncology (ESMO) International Consortium Study on the availability, out-of-pocket costs, and accessibility of antineoplastic medicines in countries outside of Europe. Annals of Oncology 28: 2633–2647, 2017. doi:10.1093/annonc/mdx521. 2017.

53. Beran, D., Ewen, M., Lipska, K., Hirsch, I. B., and Yudkin, J. S. (2018). Availability and affordability of essential medicines: implications for global diabetes treatment. Curr. Diabetes Rep. 18 (8), 48. doi: 10.1007/s11892-018-1019-z.

54. Ewen, M., Zweekhorst, M., Regeer, B., and Laing, R. (2017). Baseline assessment of WHO's target for both availability and affordability of essential medicines to treat non-communicable diseases. PloS One 12 (2), e0171284. doi: 10.1371/ journal. pone.0171284

55. Robertson J., Barr R., Shulman L.N, Forted G.B, Magrini N. Essential medicines for cancer: WHO recommendations and national priorities; 2016. Bull World Health Organ 2016; 94:735–742.

56. Barr R, Robertson J. Access to Cytotoxic Medicines by Children with Cancer: A Focus on Low- and Middle-Income Countries. Pediatric Blood Cancer 2016; 63:287–291. 2016.

57. Ministry of Health. National Health Policy: Ensuring healthy lives for all (revised edition). 2020.

58. Iyengar S, Van den Ham R, Suleman F. Medicine Price Surveys, Analyses, and Comparisons. Medicine Prices in Africa. ISBN 978-0-12-813166-4 https://doi.org/10.1016/B978-0-12-813166-4.00007-3.

Click here to access/download;Figure;Fig 2c.tiff

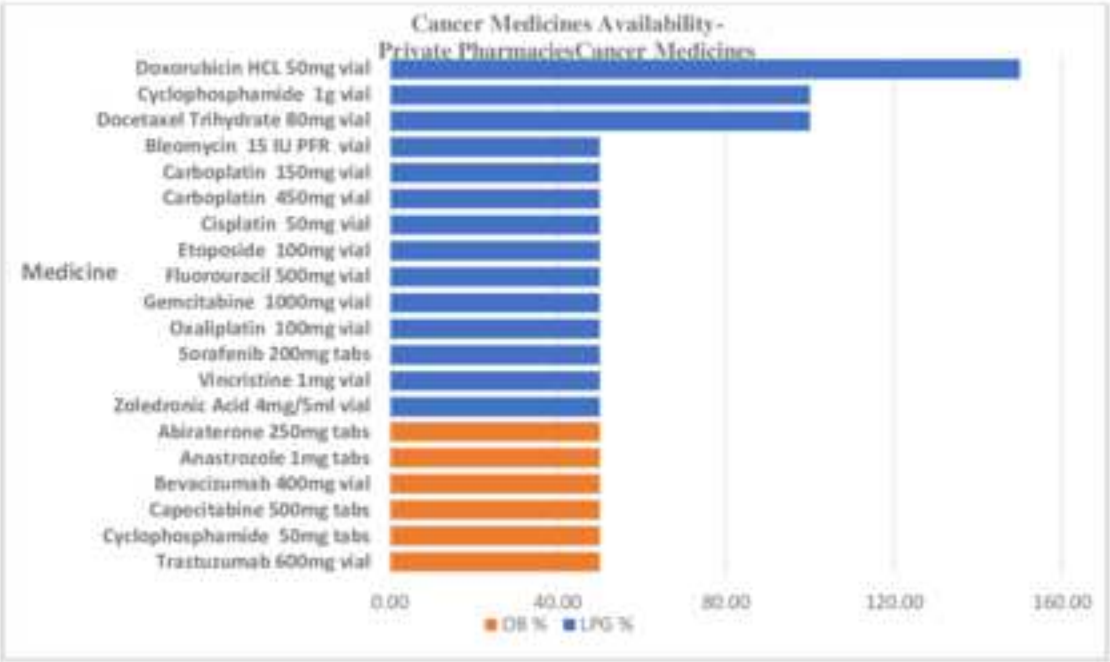

Click here to access/download;Figure;fig-10.tiff

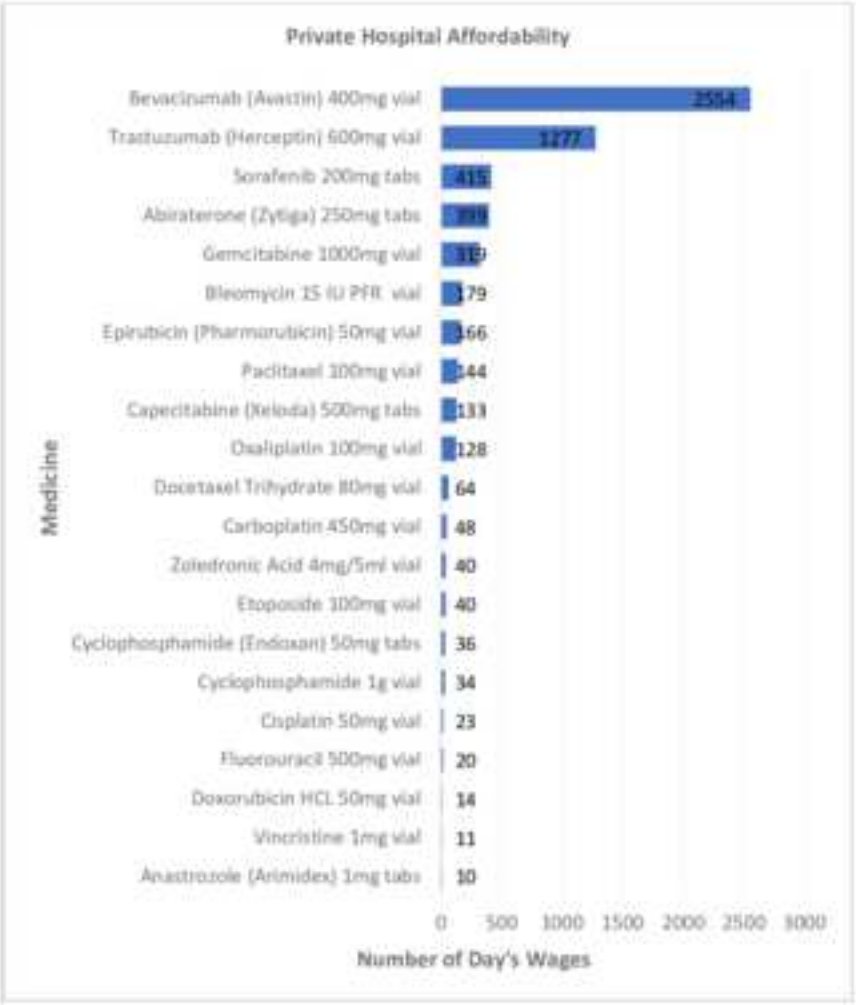

Click here to access/download;Figure;Fig-2a.tiff

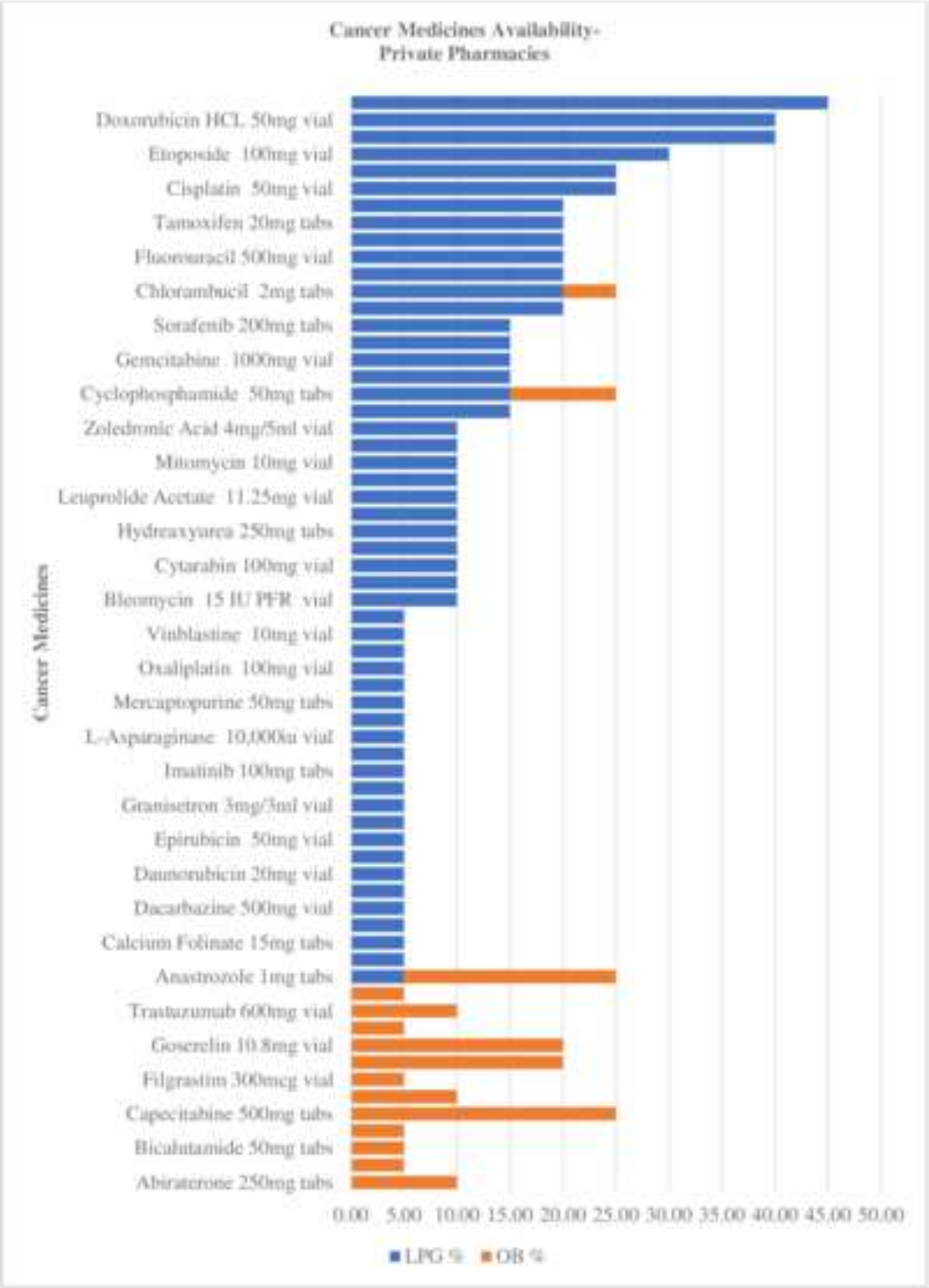

Click here to access/download;Figure;Fig-2b.tiff

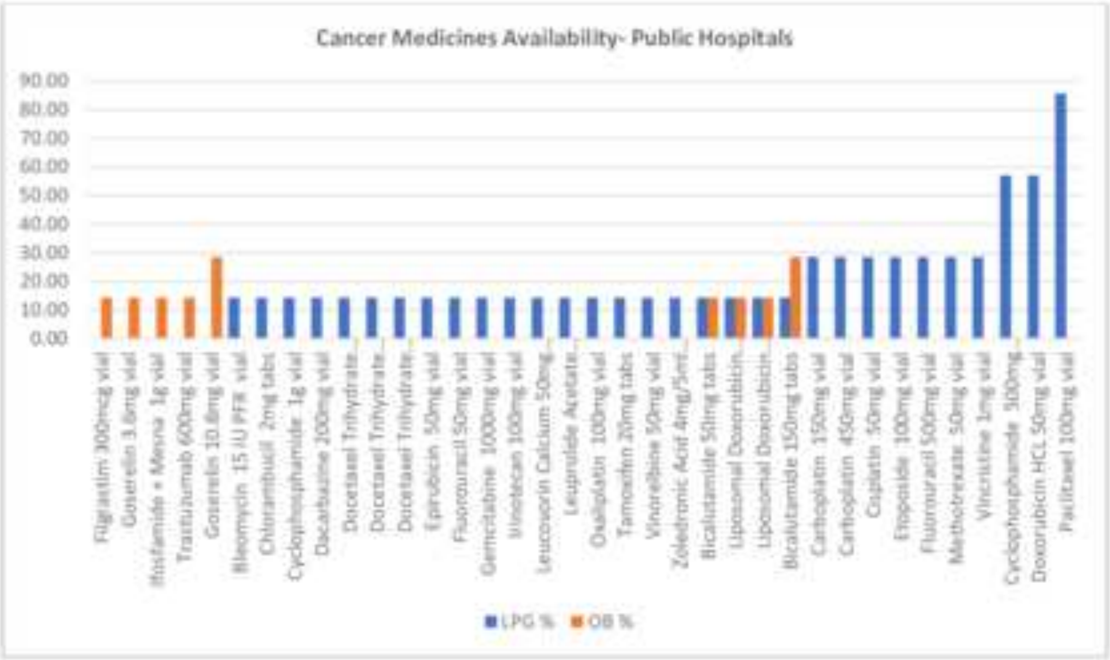

Click here to access/download;Figure;fig-4.tiff

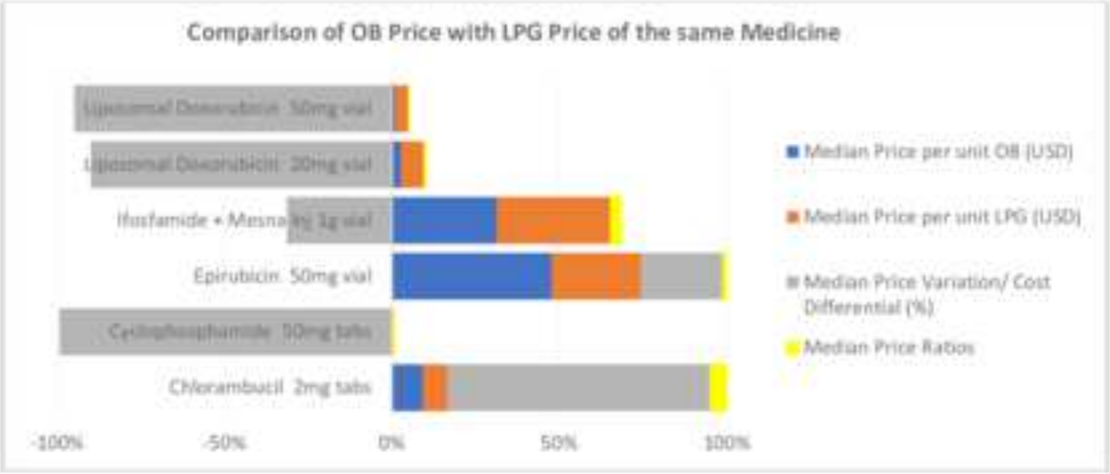

Click here to access/download;Figure;fig-5.tiff

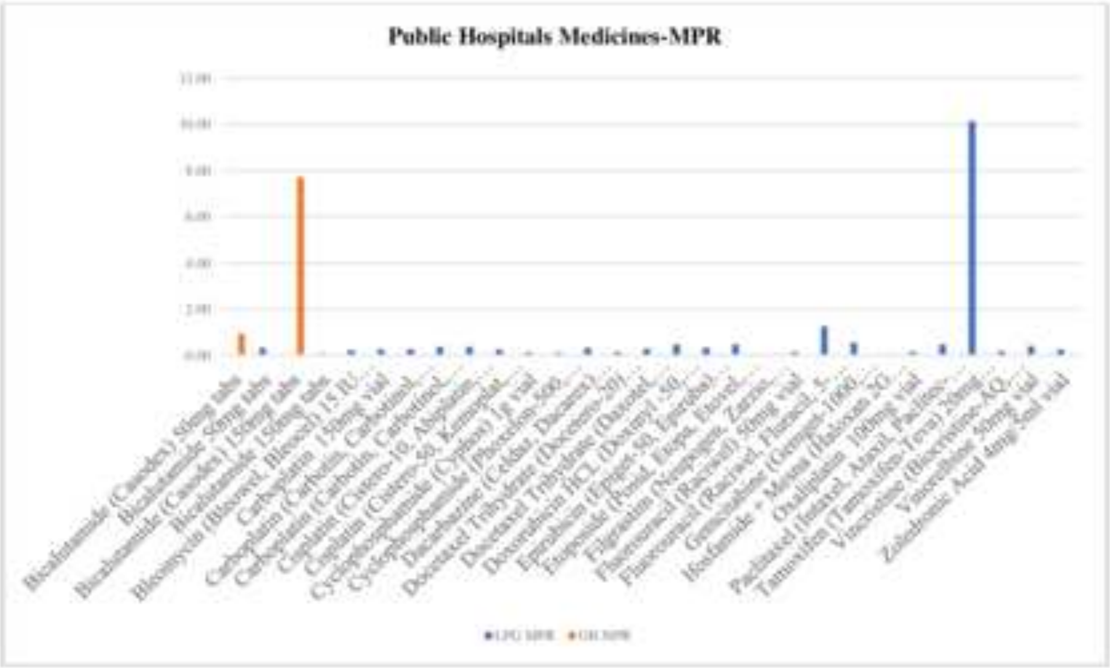

Click here to access/download;Figure;fig-8.tiff

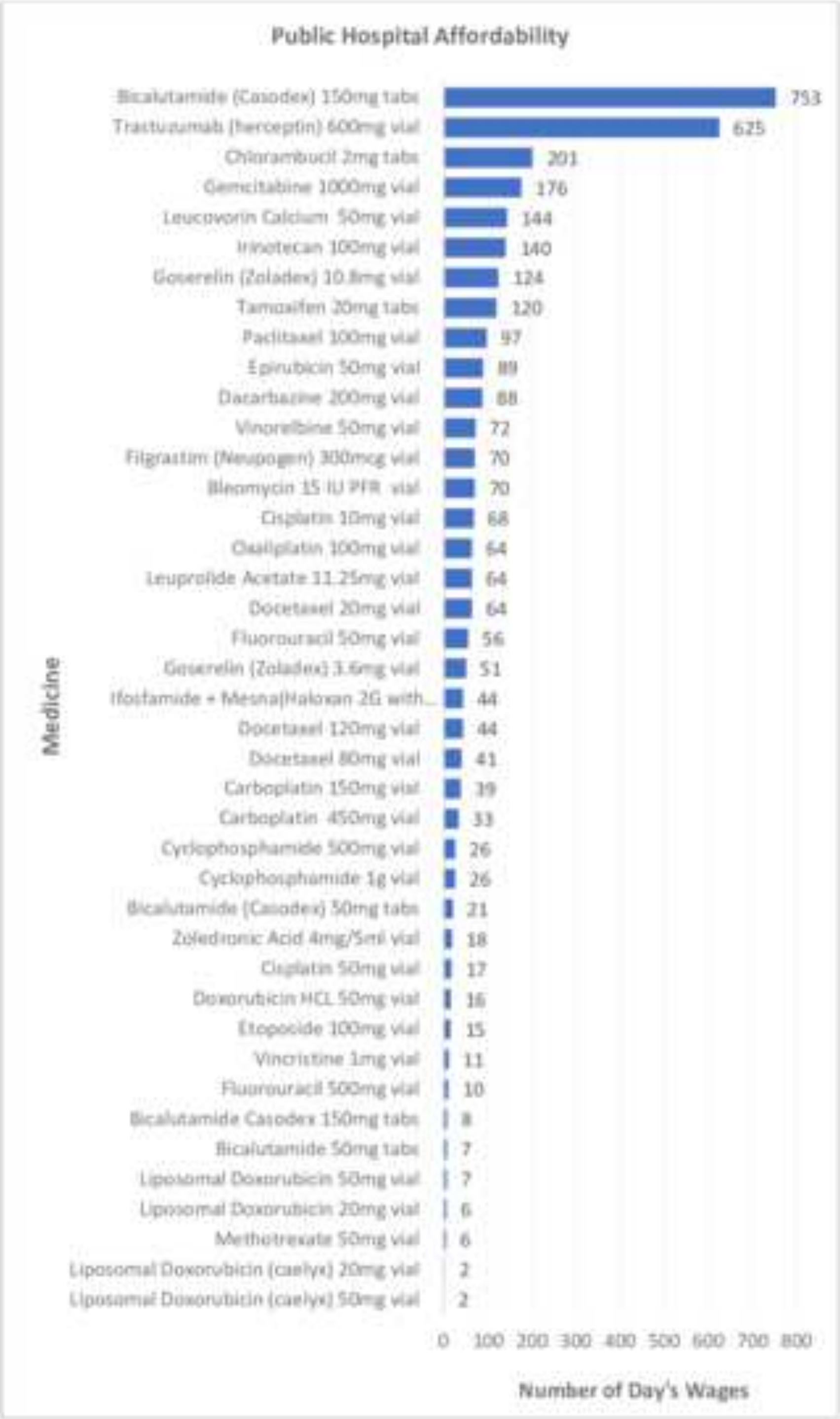

Click here to access/download;Figure;fig-9.tiff

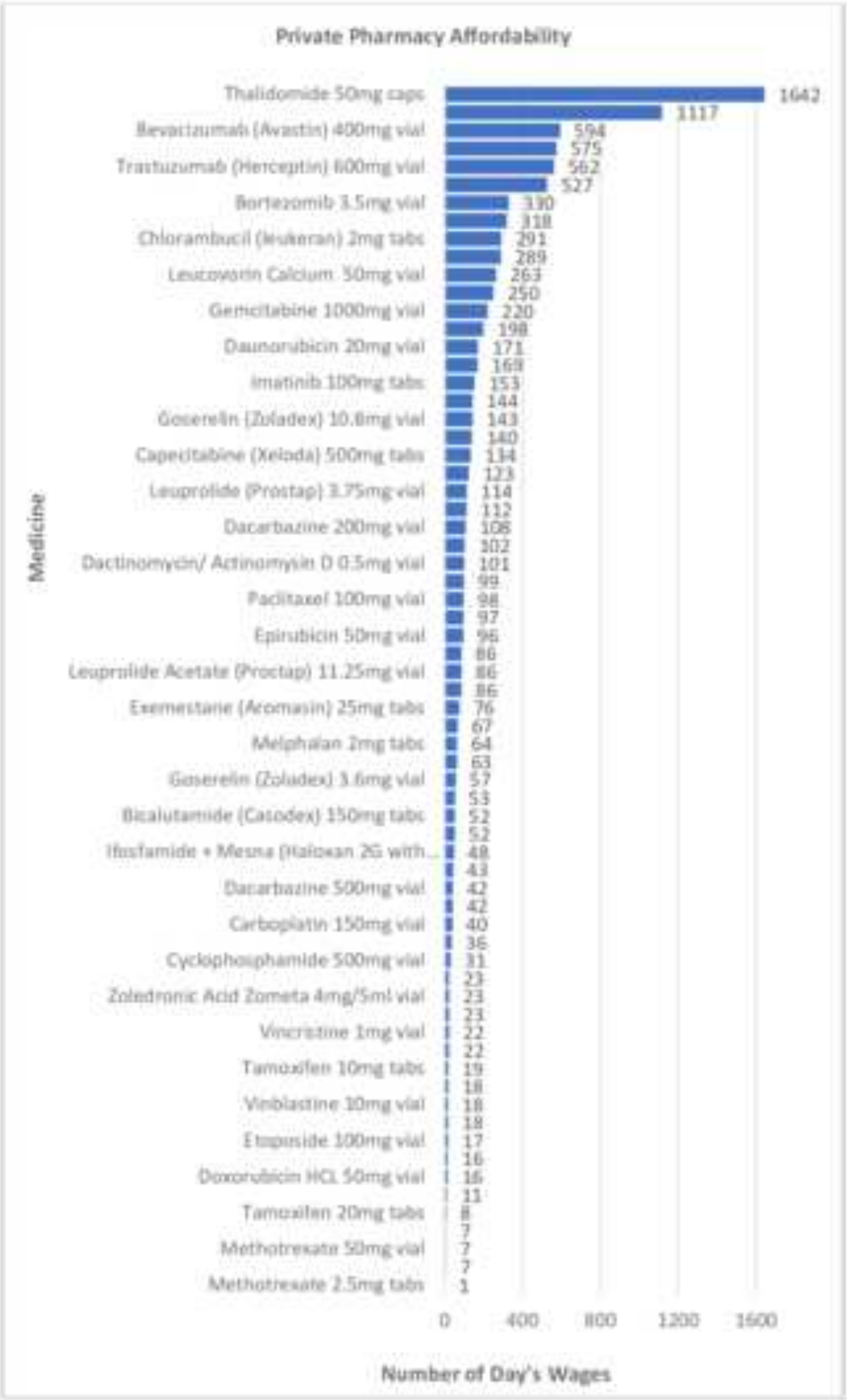

Figure Click here to access/download;Figure;fig-1-a (1).tiff

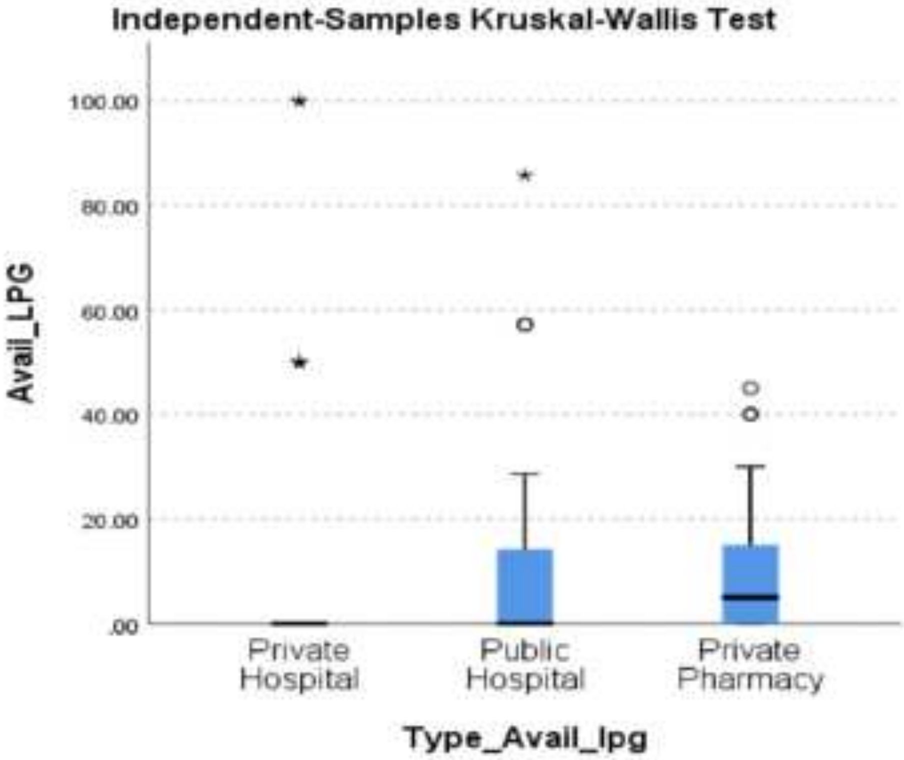

Click here to access/download;Figure;Fig_6.tiff

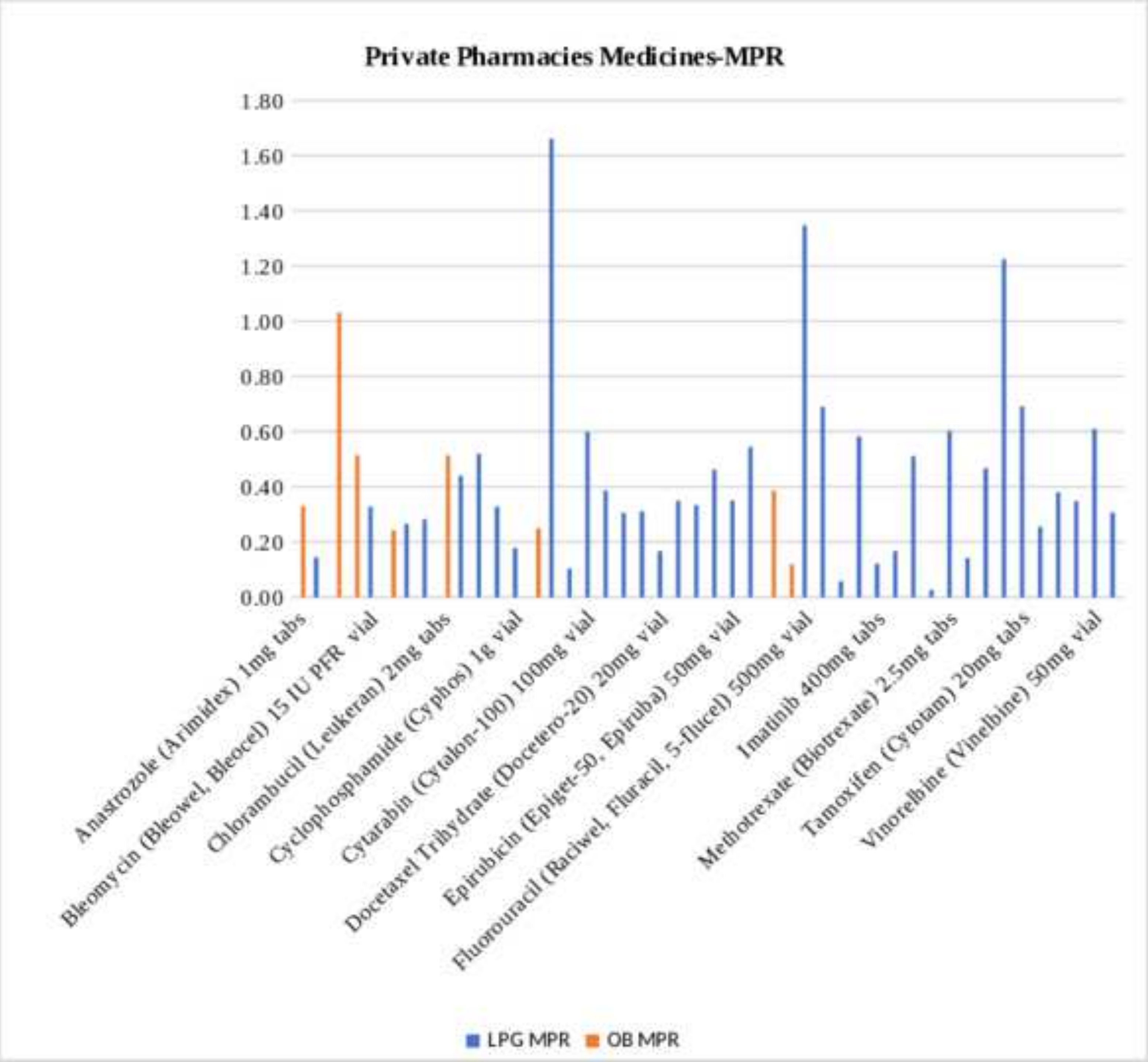

Click here to access/download;Figure;Fig-1b (1).tiff

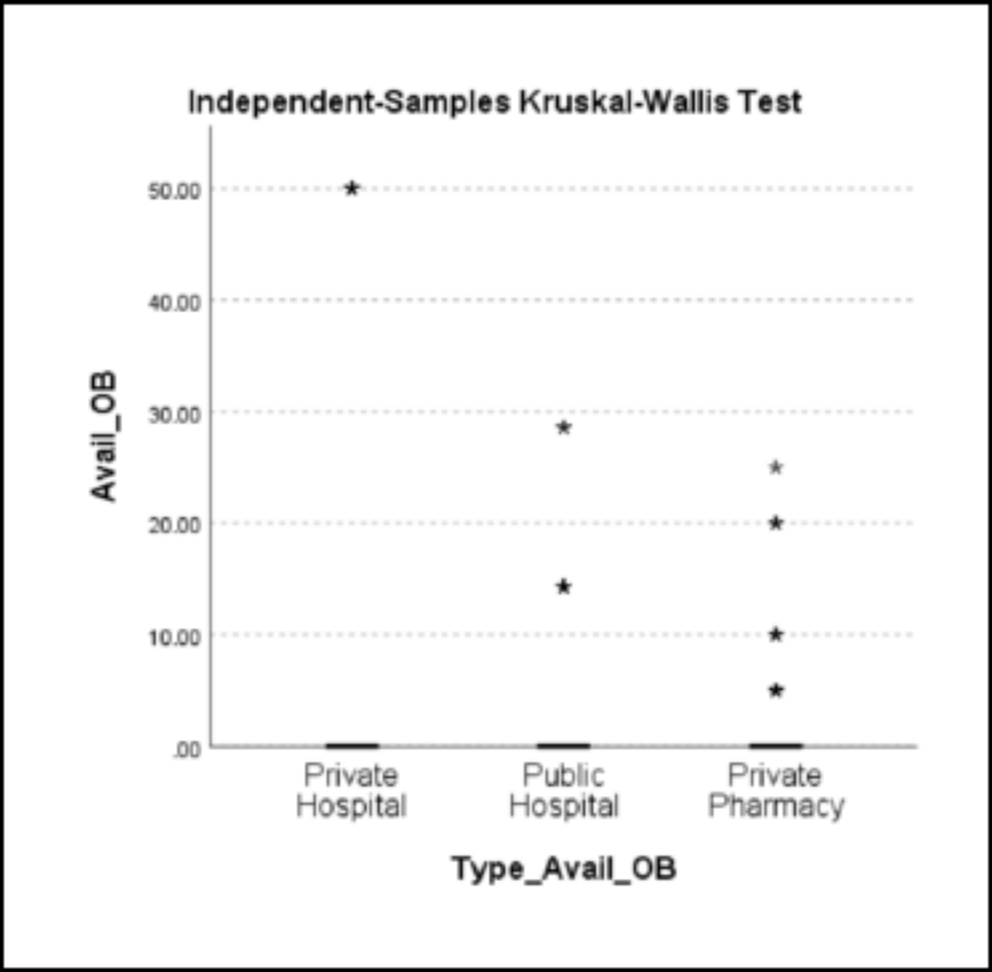

Click here to access/download;Figure;fig-3a (1).tiff

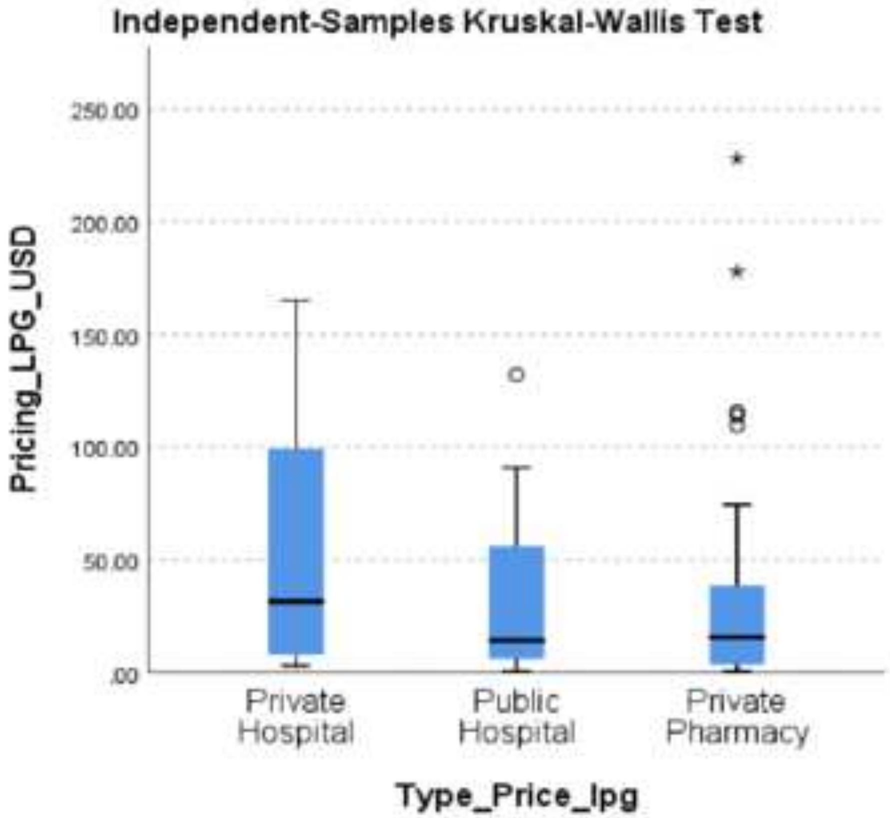

Click here to access/download;Figure;fig-3b (1).tiff

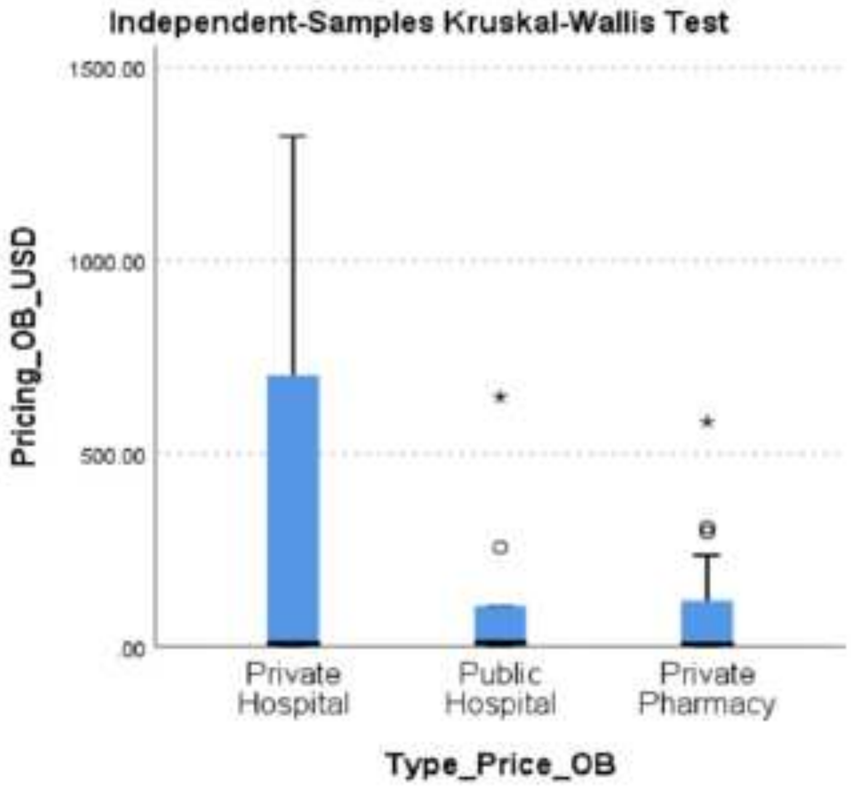

Click here to access/download;Figure;Fig-7b (1).tiff

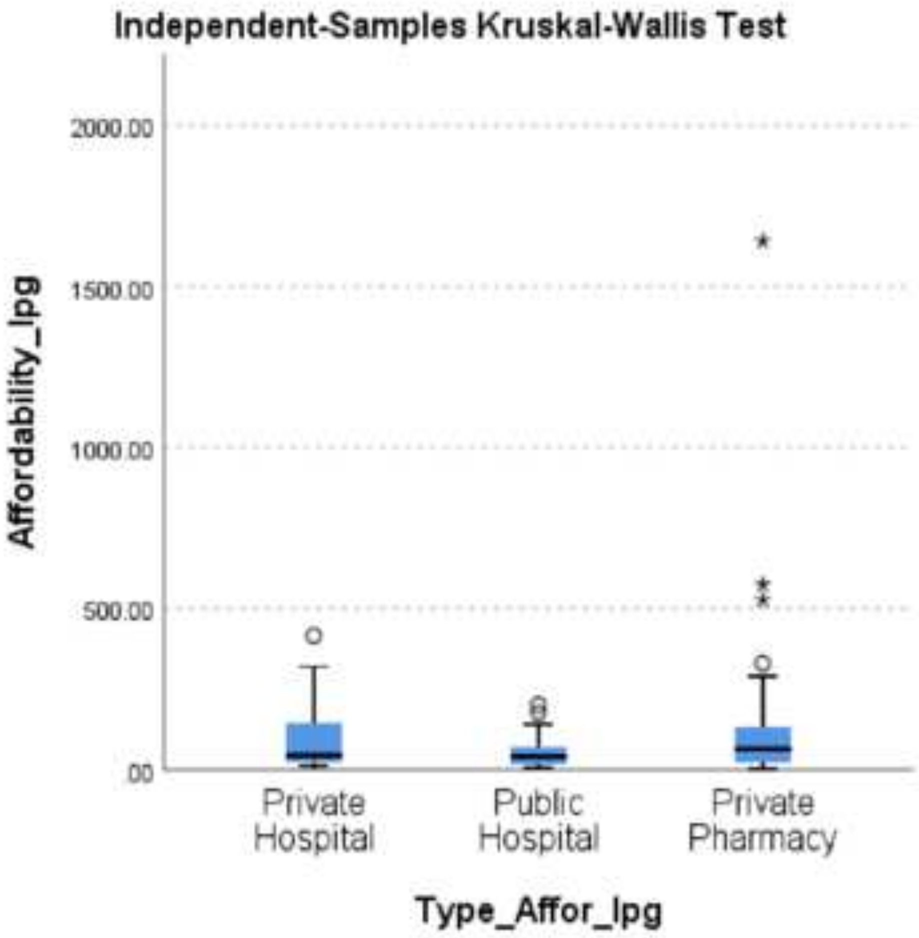

Figure

Click here to access/download;Figure;fig-7a (2).tiff

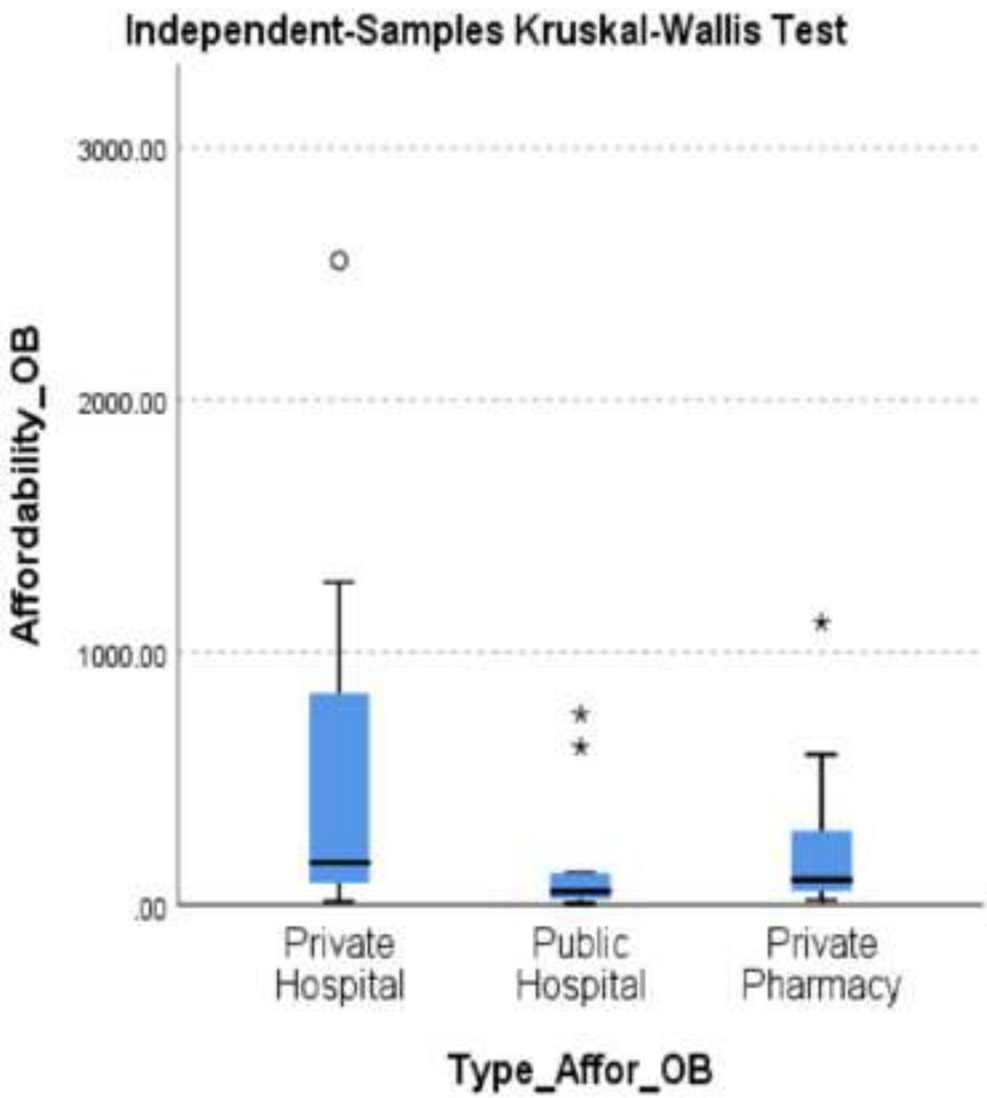

Figure
Click here to access/download;Figure;fig 7a.docx

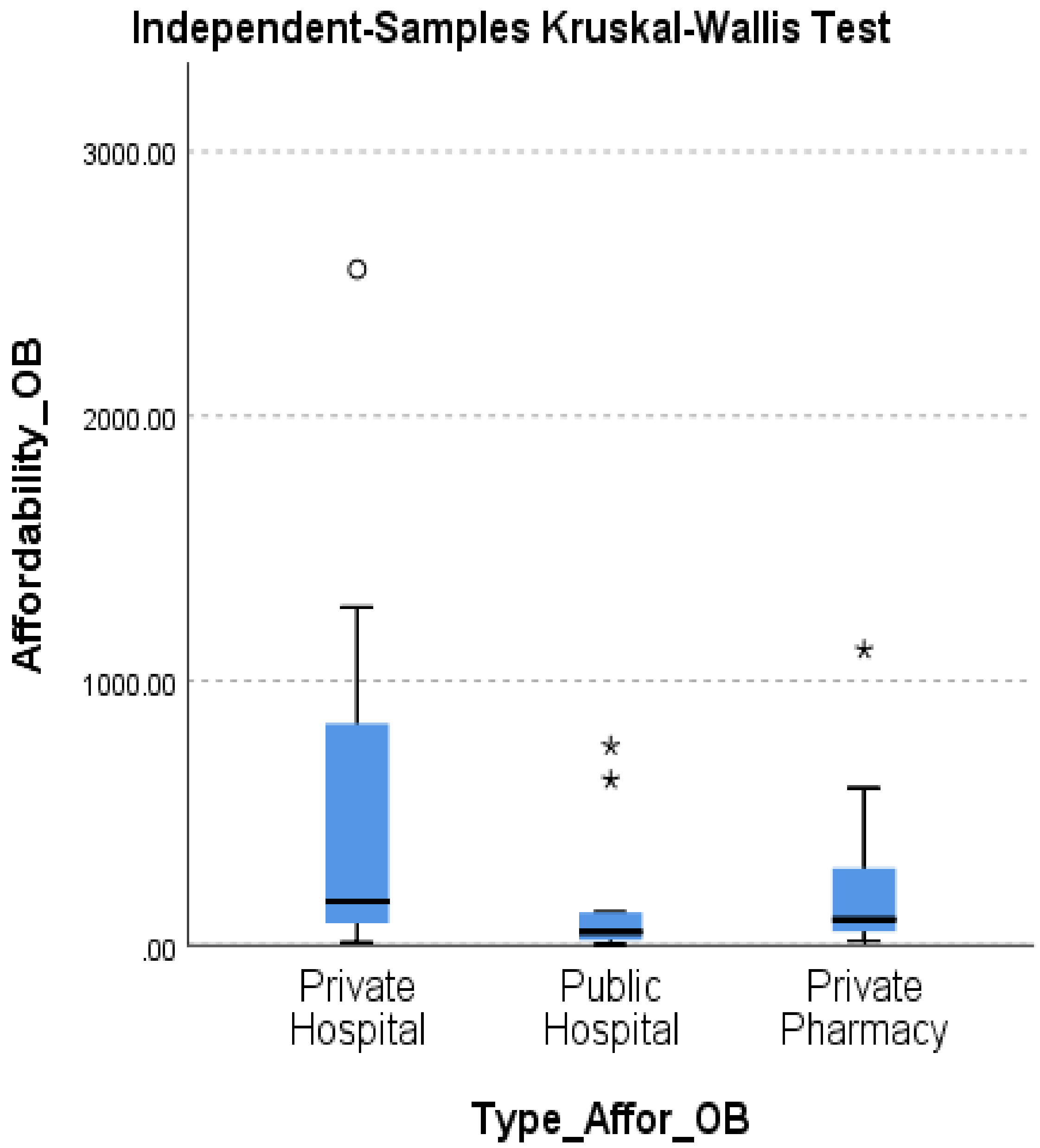

Supplement: Attachment — Submitted filename: PONE-D-21-38310_FINAL comments.pdf [file pone.0279817.s012.pdf]
