# Supplementary material for: A comprehensive survey of cancer medicines prices, availability and affordability in Ghana
Source: PLoS One. 2023 May 3;18(5):e0279817. doi: 10.1371/journal.pone.0279817 (PMC10155977; doi:10.1371/journal.pone.0279817)
Supplement: Attachment — Submitted filename: 221111 Response to Reviewers Comments.docx [file pone.0279817.s014.docx]

**Response to Reviewers’ Comments**

| **1^st^ Reviewer’s comment** | **Authors’ response** |
| --- | --- |
| Ques:  - I suggest to include a sentence in the introduction paragraph describing the objective of the study. As it stands now, it is not clear and  I would not suggest to have this sentence in the methods.    In the results section, the sentence 'Only two cancer conditions are covered by the National Health Insurance Scheme' does not seem relevant or it is disconnected to the rest. Please revise  - The reviewer suggest to refer and include this reference also: https://doi.org/10.1186/s12913-020-05167-9.  - In the methods section you introduced the abbreviations LPG, OB, MPR, etc, could you please indicate what they mean?  - Methods - please explain why you determined the procurement efficiency. What does this measure say and why is it relevant? This measurement was not found in the results, please explain and revise.  - Methods - I would suggest to revise again how affordability was calculated (you can also refer on how it was done here: https://doi.org/10.1186/s12913-020-05167-9). It is important to justify why they calculation considered one whole month of treatment, when in reality, for most cancer treatments (mainly IV treatments) are not taken daily for a month, but once every X period of time. I strongly suggest to revise this calculations and justify better. No patient would pay for 1 month of treatment (1 treatment per day for 30 days) but would pay for 1 round of treatment (for most iv medicines, this might not be the case for tablets which might be taken daily for a month and in this case, the WHO/HAI calculation would be appropriate). Because most cancer medicines are not taken daily for a month, it might be needed to adapt WHO/HAI's calculation for this reason.  For example - having treatment from bevacizumab 400mg will only require X amount of ml from the whole vial and would only be taken once in a cycle of treatment (and not daily for a month) - although I am sure it would still be unaffordable, it would be a more fair calculation to consider how much of a medicine a patient would actually use in one round of treatment to know how (un)affordable it is. (this would also be more accurate for advocacy purposes)  - Discussion - please revise the paragraph sentences 470-481. It is difficult to understand the message you want to convey and how it relates to your results.  - Discussion - I would suggest to include the reference suggested above in line 459 and 506  - Limitations - I would also add to the affordability issue that the entire cost of treatment would be even more unaffordable considering that one course of treatment requires more than once medicine, in addition to the factors already mentioned by the authors (dispensing fees, facility fees, administration and doctors fees, etc.) | Thank you for your comment. We have revised the abstract text based on your feedback.  The following reference ‘Moye-Holz, D., Ewen, M., Dreser, A. et al. Availability, prices, and affordability of selected essential cancer medicines in a middle-income country – the case of Mexico. BMC Health Serv Res 20, 424 (2020).  <https://doi.org/10.1186/s12913-020-05167-9> has been referred and included.  Abbreviations have been explained in text.  Procurement efficiency removed from text, however the difference between HPM and LPM in the results gives an indication of price variations of the same medicine. This is a sign of how efficient the procurement system is in obtaining competitive prices for the medicines.  For affordability calculations, it was based on the WHO/HAI methodology (Ref 32).  We agree with you on your comments and to note that this was taken into consideration for the calculations.  For chronic cancer illnesses, we expressed the treatment in monthly amounts, which was calculated by multiplying the daily dose by 30. For acute cancer illnesses, we considered the episodes of treatment within a month Ref: dosage column in table 8C.  The cost of a course of therapy for important conditions was compared with the daily wage of the lowest-paid unskilled government worker. This analysis is very valuable as an advocacy tool since it expresses prices in relation to an individual’s ability to pay.  Discussion: text 470-481 revised accordingly. Reference (no. 59) has been included in 459 and 506.  Limitations – text revised accordingly. |
| 2^nd^ Reviewer’s Comments |  |
| Ques:  None | None |
| 3^rd^ Reviewer’s Comments |  |
| Ques:  The authors should improve the results presentation section.  The following tables can be summarized or uploaded as supplementary files: Table 2, 5, 6 and 8.  The discussion section should show the difference between results presented in Table 3. "Percentage Availability Data using ‘mean of median’" and Figs 1a and 1b. "Percentage Availability of LPG and OB using Kruskal Wallis Test".  What is new as scientific information? Is there any king of redundancy?! Just make sure to avoid duplications, cross-check the information presented in tables against the information presented in figures. | Thank you for your comment. We have revised the text based on your feedback.  These tables are now uploaded as supplementary files.  The percentage availability data using Kruskal Wallis test in Figs 1a and 1b is shown to complement Table 3. |
| 4^th^ Reviewer’s Comments: |  |
| Ques:  The English of the article, while understandable, does contain grammatical and spelling errors. I suggest the authors to check and correct these errors carefully. | Thank you for your comments. We have revised accordingly. |
| 5^th^ Reviewers Comments: |  |
| Ques: I have only one concern. Generally, most cancer medicines are restricted drugs. So I am quite surprised to know that cancer medicines, both tablet/capsule and injection can be sold in pharmacies in Ghana.  My further question is that do they need prescription? Therefore, it might be useful if authors could provide more detail on how cancer medicines are controlled and dispensed in pharmacies in Ghana. | Thank you for your comments. Text has been added to show that cancer medicines are only dispensed when prescribed and obtained by prescription, thus controlling its use as restricted drugs. |
| Editor Comments:  Please review your reference list to ensure that it is complete and correct. If you have cited papers that have been retracted, please include the rationale for doing so in the manuscript text, or remove these references and replace them with relevant current references. Any changes to the reference list should be mentioned in the rebuttal letter that accompanies your revised manuscript. If you need to cite a retracted article, indicate the article’s retracted status in the References list and also include a citation and full reference for the retraction notice. | Thank you for your comment. This has been revised according to editors’ suggestions. |
